# Sediment bacterial biogeography across reservoirs in the Hanjiang river basin, southern China: the predominant influence of eutrophication-induced carbon enrichment

**DOI:** 10.3389/fmicb.2025.1554914

**Published:** 2025-03-28

**Authors:** Haokun Yang, Xueling Xiong, Yiping Tai, Li-Juan Xiao, Dan He, Liqin Wu, Lijun Zhou, Lijuan Ren, Qinglong L. Wu, Bo-Ping Han

**Affiliations:** ^1^Department of Ecology and Institute of Hydrobiology, Jinan University, Guangzhou, China; ^2^Center for Evolution and Conservation Biology, Southern Marine Sciences and Engineering Guangdong Laboratory (Guangzhou), Guangzhou, China; ^3^School of Environmental Science and Engineering, Guangzhou University, Guangzhou, Guangdong, China; ^4^State Key Laboratory of Lake Science and Environment, Nanjing Institute of Geography and Limnology, Chinese Academy of Sciences, Nanjing, China

**Keywords:** sediment bacteria, subtropical reservoirs, biogeographic pattern, niche breadth, homogeneous selection

## Abstract

A fundamental goal of reservoir ecosystem management is to understand bacterial biogeographic patterns and the mechanisms shaping them at a regional scale. However, little is known about how eutrophication, a major water quality challenge in reservoirs, influences sediment bacterial biogeographic patterns in subtropical regions. In this study, sediment bacterial communities were sampled from 21 subtropical reservoirs in the Hanjiang river basin, southern China, and spanning trophic states from oligotrophic to eutrophic. Our findings demonstrated that eutrophication-driven changes in total carbon (TC) significantly shaped the regional biogeographic patterns of sediment bacterial communities, weakening the “distance-decay” relationships that typically link bacterial community similarity to geographical distance. TC content exceeding a threshold of 13.2 g·kg^−1^ resulted in substantial shifts in bacterial community structure. Specifically, high TC levels promoted the dominance of copiotrophic bacteria such as *Syntrophales* (Deltaproteobacteria), *Clostridiaceae* (Firmicutes), and VadinHA17 (Bacteroidetes), while oligotrophic taxa like *Anaerolineaceae* (Chloroflexi) and Nitrospirota were prevalent in low TC sediments. Additionally, higher TC content was associated with increased regional heterogeneity in bacterial community composition. Reservoirs with elevated TC levels exhibited more complex bacterial interaction networks, characterized by stronger niche segregation and higher competition compared to low TC networks. Overall, these findings underscore the pivotal role of sediment TC in shaping bacterial biogeography at a regional scale. They provide valuable insights for predicting ecosystem responses to eutrophication and offer guidance for mitigating the impacts of anthropogenic activities on freshwater ecosystems.

## 1 Introduction

Understanding biogeography and the underlying mechanisms are fundamental objectives of freshwater ecology (Abell et al., 2008; Xu et al., 2020; Amend et al., 2022). Unlike natural freshwater ecosystems, such as lakes and rivers, reservoirs are artificial systems formed by damming rivers or valleys (Nilsson and Berggren, 2000). They serve multiple purposes, supporting both economic and ecological development through water storage and supply, flood control, agricultural irrigation, hydroelectric power generation, and climate regulation (Reitter et al., 2021). The biogeographic patterns observed in reservoirs arise from a dynamic interplay between deterministic and stochastic factors, including environmental filtering, random dispersal events, and geographic isolation (Gittins et al., 2023). While spatial proximity often correlates with microbial community similarity due to shared environmental conditions and historical contingencies (Liu et al., 2015; Gittins et al., 2023), anthropogenic inputs of nitrogen (N) and phosphorus (P) have triggered widespread eutrophication in aquatic systems (Smith, 2003; Smith and Schindler, 2009; Yang et al., 2012). This nutrient enrichment has led to divergent trophic states ranging from oligotrophic to hypereutrophic among neighboring reservoirs. Consequently, shifts in microbial community structure driven by trophic state gradients increasingly supersede geographic distance as the dominant driver of biogeographic variation (Liu et al., 2019; Tang et al., 2019).

Eutrophication profoundly impacts the carbon cycle in reservoir ecosystems, driving the accumulation of total carbon (TC) in sediments (Smith and Schindler, 2009; Yang et al., 2012). Sediment bacterial communities respond quickly to TC deposition, making them valuable indicators of environmental change (Sun et al., 2012). These microbes play a crucial role in metabolic processes, breaking down complex organic polymers (e.g., cellulose and chitin) through extracellular enzymes (e.g., cellulases and chitinases), thereby releasing bioavailable carbon, nitrogen, and phosphorus into the water column. This process supports primary production and sustains higher trophic levels (Buchan et al., 2014; Sinsabaugh et al., 2014; Sun et al., 2012; Wang et al., 2020). Despite growing awareness of eutrophication's influence on bacterial communities, critical knowledge gaps persist regarding how eutrophication-driven TC accumulation shapes bacterial biogeography across different trophic states and reservoir types. Specifically, the effects of TC variation on bacterial diversity, ecological interactions, and network structure in reservoirs with varying nutrient conditions remain poorly understood. Additionally, while TC enrichment is known to influence bacterial metabolic strategies, its role in shifting the balance between deterministic and stochastic processes in bacterial community assembly has yet to be fully explored. Addressing these gaps is essential for advancing our understanding of bacterial ecology in eutrophic aquatic systems and informing effective reservoir management strategies in response to increasing nutrient inputs at a regional scale (Yang et al., 2021).

The diversity of bacteria in reservoir sediments is closely linked to the amount of total organic carbon present (Filippini et al., 2019; Thomas et al., 2020). Variations in total organic carbon deposition can also influence bacterial ecological interactions in sediments (Säwström et al., 2016). Since eutrophication-driven organic carbon accumulation directly contributes to total carbon levels, variations in total carbon serve as an indicator of both anthropogenic nutrient inputs and their ecological consequences. For example, increased nitrogen and phosphorus concentrations can promote heterotrophic bacteria that metabolize labile carbon substrates (e.g., Flavobacterium and Bacteroidetes), while suppressing oligotrophic taxa (e.g., Acidobacteria) that thrive in low-nutrient conditions (Newton et al., 2011; Liu et al., 2019). Variations in total carbon deposition can also influence bacterial correlation patterns and ecological interactions in sediments (Säwström et al., 2016). Bacterial communities display intricate correlation patterns, indicative of complex ecological interactions and connectivity within the community (Faust and Raes, 2012; Zhang et al., 2018; Wang et al., 2023; Yu et al., 2023). Metabolic cross-feeding, where byproducts of one taxon (e.g., fermentation-derived acetate) serve as substrates for others (e.g., sulfate-reducing Desulfobacter), further drives these interactions (D'Souza et al., 2018). Some bacterial taxa co-occur due to their shared ability to metabolize specific carbon compounds, while others partition carbon resources to minimize competition (Liu et al., 2019; Tang et al., 2019). The correlation relationships can be visualized as networks, where bacterial taxa are represented as “nodes,” and their interactions as “links,” revealing clusters of co-occurred taxa responding to environmental carbon changes (PrŽulj and Malod-Dognin, 2016; Layeghifard et al., 2017; Liu et al., 2021; Wallenius et al., 2021). However, the ways in which eutrophication influences the correlation patterns and network modules of sediment bacterial communities at a regional scale remain poorly understood.

Additionally, eutrophication-driven changes may modify the balance between deterministic and stochastic processes underlying bacterial biogeography (Stegen et al., 2012; Pearson et al., 2018; Huber et al., 2020). Changes in sediment nutrient conditions may drive convergence or divergence in bacterial community compositions at a regional scale, depending on their local community assembly processes (Zeng et al., 2019; Zuo et al., 2024). Deterministic processes suggest that bacterial biogeography is driven by environmental heterogeneity or ecological interactions (Zhou and Ning, 2017). In contrast, stochastic processes assume that all bacterial taxa are ecologically and functionally equivalent, and their distributions are independent of environmental conditions (Stegen et al., 2012; Chen et al., 2019). Stochastic processes, such as random dispersal and ecological drift, can facilitate the establishment of rare bacterial taxa, contribute to community heterogeneity, and alter bacterial biogeography at regional scales (Wang et al., 2024). Anthropogenic activities such as dam construction impact reservoir ecosystems by forming dispersal barriers, isolating reservoirs with diverse hydrological and geographical conditions (Zarri et al., 2022). These dispersal barriers interact with eutrophication-driven carbon changes, potentially preventing homogenization of bacterial communities across reservoirs and increasing the role of both local environmental filtering and regional dispersal limitation in bacterial assembly (Chase and Ryberg, 2004; Chen et al., 2019; Huber et al., 2020; Pinto et al., 2021). Despite the ecological importance of community assembly processes, the relative contributions of deterministic and stochastic factors in shaping bacterial biogeography remain insufficiently understood, especially in subtropical reservoirs.

This study investigated sediment bacterial biogeography and its underlying mechanisms in subtropical reservoirs of the Hanjiang river basin, southern China. These reservoirs, heavily influenced by anthropogenic nutrient inputs, present a valuable yet underexplored context for understanding sediment bacterial biogeography and their responses to eutrophication (Han and Dumont, 2011; Liu et al., 2019; Nyirabuhoro et al., 2020). We hypothesized that: (1) eutrophication-induced carbon changes in geographically proximate reservoirs might disrupt the expected similarity of bacterial community compositions due to geographic proximity, and reshape bacterial biogeographic patterns on a broader regional scale; and (2) the relative contributions of deterministic vs. stochastic processes in shaping bacterial correlation patterns and biogeography varied with changes in trophic states. Our findings may offer critical insights into the biogeographic patterns of sediment bacterial communities under eutrophication, informing strategies for effective reservoir management and conservation as nutrient loads continue to rise regionally.

## 2 Materials and methods

### 2.1 Sample collection

**Figure 1:**
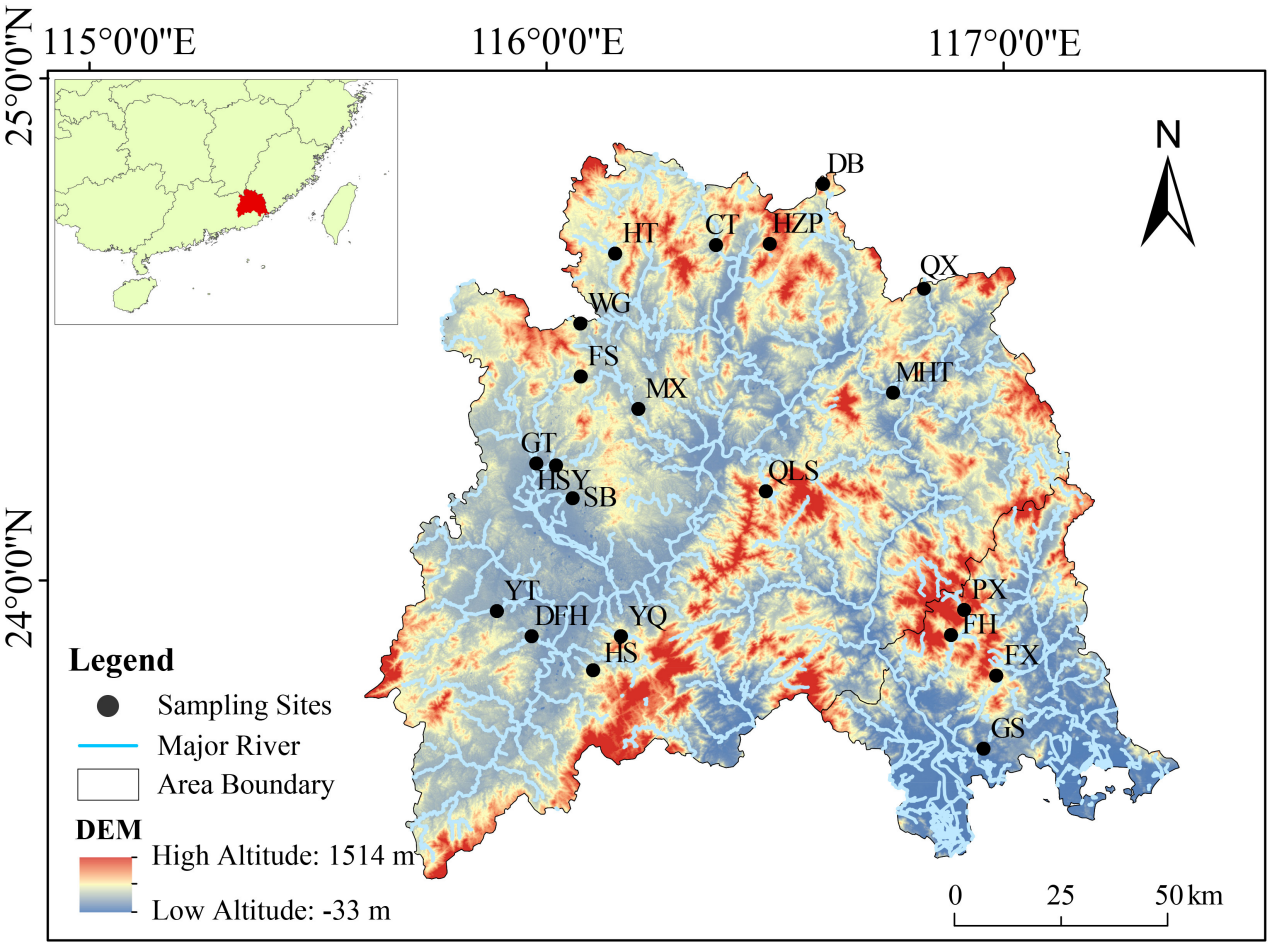
Location of sampling sites in 21 reservoirs in the Hanjiang river basin, southern China. CT, Changtan reservoir; DB, Duobao reservoir; DFH, Dongfanghong reservoir; FH, Fenghuang reservoir; FS, Fushi reservoir; FX, Fengxi reservoir; GS, Gangshan reservoir; GT, Guitian reservoir; HS, Heshui reservoir; HSY, Heshanyan reservoir; HT, Huangtian reservoir; HZP, Huangzhuping reservoir; MHT, Mianhuatan reservoir; MX, Meixi reservoir; PX, Pengxi reservoir; QLS, Qingliangshan reservoir; QX, Qingxi reservoir; SB, Shibi reservoir; WG, Wengong reservoir; YQ, Yanqian reservoir; YT, Yitang reservoir.

From November 2019 to January 2020, a total of 63 sediment samples were collected from 21 reservoirs in the Hanjiang river basin in Guangdong Province, China (E 115°13′-117°09′, N 23°17′-26°05′; Figure 1). The Hanjiang river basin, covering an area of 3.01 × 10^4^ km^2^, is the second-largest watershed in Guangdong Province. The study included 21 reservoirs, comprising three large reservoirs and 18 medium-sized ones. To ensure broad geographic coverage and minimize potential bias, the sampling sites were selected to encompass reservoirs with varying nutrient levels, land use impacts, and hydro-morphological characteristics. Within each reservoir, three sediment samples were collected from the central zone, ~10 m apart. This area was chosen to provide a more stable and representative record of sediment deposition, as it is less affected by localized disturbances such as shoreline erosion and inflow fluctuations compared to nearshore regions.

The Hanjiang river basin has a subtropical climate, with annual precipitation ranging from 1,500 to 2,000 mm, which varies across the basin. Most reservoirs in the region function as rain catchments, exhibiting strong directional water flow, and being heavily influenced by anthropogenic activities, such as agricultural runoff, industrial pollution, and urban development (Lin and Han, 2001). These factors play a critical role in shaping water quality and nutrient dynamics in the reservoirs. Sampling was conducted during the dry season (November 2019–January 2020) to minimize the effects of seasonal hydrological fluctuations, such as heavy rainfall and flooding, which could alter sediment composition and microbial community structure. This period was chosen to provide a more stable representation of long-term organic matter accumulation and nutrient deposition rather than transient seasonal variations. Sediment cores were vertically collected from the central zone of each reservoir using a Uwitec columnar sediment sampler (PVC tube: 60 cm length, 6 cm diameter), targeting a depth of 10–30 cm. This depth range was chosen to minimize the influence of surface disturbances and seasonal variability, providing a historical snapshot of organic matter deposition and microbial community structure (Nelson and Sommers, 1982; Wan et al., 2017). Water depths in the reservoirs ranged from 5 to 50 m. Each sediment sample was carefully cleaned of extraneous matter, thoroughly homogenized, sealed in 50 ml sterilized polyethylene tubes, and placed in a cooling box for transport. Samples were then stored at −80°C in the laboratory for subsequent DNA extraction and analysis.

### 2.2 Physicochemical characterization analyses

To analyze the main physicochemical properties of the sediment samples, sediments were placed in Petri dishes and frozen at −30°C for 24 h. The samples were then freeze-dried for 48 h, ground using an onyx mortar, and sieved through a 100-mesh sieve. The sediment mass before and after drying was recorded, and sediment-water content (SWC) was calculated using the drying constant weight method.

Sediment total nitrogen (TN) and total phosphorus (TP) were determined using the persulfate digestion method (De Borba et al., 2014). Sediment total carbon (TC), total hydrogen (TH), and total sulfur (TS) were measured with an elemental analyzer (EA2400II, PE Inc., USA). The dissolved magnesium (Mg^2+^), calcium (Ca^2+^), iron (Fe^3+^), copper (Cu^2+^), and manganese (Mn^2+^) in the sediments were analyzed using graphite furnace atomic absorption spectrophotometry. Additionally, the water environmental properties of the studied reservoirs were assessed. Water depth was measured on-site using a depth sounder. Water transparency (SD) was measured on-site using a Secchi disk, while nutrient analyses for total nitrogen (TN), total phosphorus (TP), and chlorophyll *a* (Chla) in the water were conducted following standard methods for water quality monitoring (Greenberg et al., 1988). The trophic status index (TSI) for each reservoir was calculated based on the concentrations of water TP, TN, Chla, and SD (Carlson, 1977).

### 2.3 DNA extraction, PCR amplification, and sequencing

Genomic DNA was extracted from the samples using a DNA extraction kit (MoBio Laboratories, Carlsbad, CA, USA) and purified with a DNA purification kit from the same manufacturer. The purified DNA served as the template for PCR amplification. Specific primers with barcodes and high-fidelity enzymes were selected for amplification. The V4 region (~291 bp) of the bacterial 16S rRNA gene was amplified using the universal primers F515 (5′-GTGCCAGCMGCCGCGG-3′) and R806 (5′-TAATCTWTGGGVHCATCAG-3′). The PCR reaction mixture consisted of 25 μl of 2 × PCR Premix Taq, 10 mM primers, 60 ng of genomic DNA, and 20 μl of nuclease-free water. The PCR cycling conditions were as follows: initial denaturation at 94°C for 5 min; 30 cycles of 94°C for 30 s (denaturation), 52°C for 30 s (annealing), and 72°C for 30 s (extension); followed by a final extension at 72°C for 10 min. The amplified products were combined in equal mass, thoroughly mixed, and verified by agarose gel electrophoresis. The target bands were extracted using a Qiagen Gel Recovery Kit. Library preparation was performed using the TruSeq^®^ DNA PCR-Free Sample Preparation Kit following the standard protocol, and the finalized library was sequenced on the Illumina HiSeq 2500 platform.

### 2.4 Data analyses

Using the barcodes and PCR amplification primer sequences, we performed the split_libraries_fastqc.py script in QIIME (v1.9.1) to demultiplex the raw reads for each sample. Sequence trimming was performed using Trimmomatic (v0.36), with the base quality threshold set to 20 for both ends of the reads (Bolger et al., 2014). FastQC was utilized to visualize the quality of the filtered reads. Paired-end reads were merged using the Vsearch plugin in QIIME2, and redundant sequences were dereplicated to generate representative sequences. Sequences were clustered into operational taxonomic units (OTUs) at 97% similarity using the cluster-features-de-novo method (Edgar, 2013). The OTU table and representative sequences were compared with the species database via Vsearch, and chimeric sequences were removed to obtain the final valid data set. Taxonomic annotation was performed using Mothur (v1.45.0) by aligning sequences against the SILVA SSU rRNA database (release 138) at the recommended bootstrap threshold of 80% (Quast et al., 2012). All sequences identified as chloroplasts and mitochondria were removed before downstream analyses. Representative sequences were aligned with MAFFT (v7.463), and maximum likelihood estimation (MLE) was applied to construct phylogenetic trees using FastTree (v2.1.10). For downstream analysis, the “phyloseq” package in R was used to normalize the data for subsequent analyses.

### 2.5 Construction of correlation networks and detection of keystones

Correlation networks were constructed to explore the relationships in complex bacterial communities under different sediment carbon levels (Meziti et al., 2016). The top 1,000 most abundant OTUs, present in more than half of the samples, were selected for correlation network analysis. In a correlation network, nodes represent different OTUs, while edges denote strong correlations between OTUs (|*R*| > 0.7, *p* < 0.05). The *p*-values were adjusted using the Benjamini–Hochberg false discovery rate (FDR) corrections. Edges with adjusted *p*-values below 0.05 and scores above the threshold determined by the random matrix theory (RMT) method were retained. Network topological properties, including average path distance, clustering coefficient, average degree, positive edge ratio, network centralization, density, heterogeneity, and connectivity as well as modularity, were calculated using the “igraph” package in R. To compare the stability of different networks, we calculated the robustness index, defined as the proportion of the remaining species in the network following node removal. The vulnerability of each node, measuring the relative contribution of the node to the global efficiency, was also estimated, and the maximal value of the nodes in a network was the network vulnerability (Yuan et al., 2021). The “ggClusterNet” package was used to calculate within-module connectivity (Zi) and among-module connectivity (Pi), with node classification thresholds set at Zi ≥ 2.5 and Pi ≥ 0.62. Finally, the network was visualized using Gephi (https://gephi.org/).

### 2.6 Statistical analyses

ArcGIS was used to visualize the sampling site distributions in the studied area. In the “vegan” package of R, we conducted the following analyses: (1) multiple α-diversity indices, including OTU richness, Shannon index, Simpson index, Pielou's evenness, Chao1, ACE, and Coverage, to evaluate bacterial community diversity across samples; (2) Non-metric Multidimensional Scaling (NMDS) to visualize bacterial community distributions based on both sampling sites and sediment total carbon (TC) levels; (3) Permutational Multivariate Analysis of Variance (PERMANOVA) to assess significant differences in bacterial community composition between different carbon groups (Anderson, 2014); (4) Principal Component Analysis (PCA) to explore the relationships among environmental variables. Prior to analysis, all environmental variables (sediment TC, TN, TP, SWC, TS, TH, Ca^2+^, Fe^3+^, Cu^2+^, Mn^2+^, Mg^2+^, water TSI, Chla, TN, and TP) were log-transformed) to reduce skewness and standardized using Z-score normalization (mean-centered and scaled to unit variance). PCA was performed using the “vegan” package in R, and the significance of principal components was assessed through permutation tests (999 iterations). (5) Canonical Correspondence Analysis (CCA) was applied to assess the relationship between bacterial community composition and sediment environmental variables. To mitigate the influence of dominant taxa, bacterial abundance data were Hellinger-transformed. Environmental variables were selected based on variance inflation factors (VIF < 10) and stepwise selection methods. The most influential abiotic nutrient variables (TP, TN, SWC, and TC) that significantly contributed to bacterial community variation were identified. The significance of the ordination was tested using ANOVA-like permutation tests (999 permutations); (6) Variance Partitioning Analysis (VPA) was performed to partition the variation in bacterial community data with respect to environmental factors (env: TC, TP, TN and SWC), metal ions (Metals: Ca^2+^, Mg^2+^, Fe^3+^, Cu^2+^, and Mn^2+^), and geographical parameters using adjusted R-squared in CCA. (7) We carried out mantel tests between transformed environmental variables with both taxonomic composition (Bray-Curtis's dissimilarity) and phylogenetic composition (UniFrac's dissimilarity); (8) We conducted multivariate regression tree (MRT) analysis to assess the significance of environmental variables in shaping bacterial community assembly. To prevent overfitting, pruning was applied using cross-validation, and the optimal tree size was selected by minimizing the relative error.

Threshold indicator taxa analysis (TITAN) was carried out to detect potential environmental threshold points driving community changes using the “TITAN2” package in R (Figary et al., 2022). The relative abundances of major bacterial lineages or clades within the top 30 OTUs were visualized along a total carbon gradient using the “pheatmap” package in R. Significant differences of bacterial dominant phyla were analyzed between different carbon groups by STAMP software (Parks and Beiko, 2010). The infer community assembly mechanisms by phylogenetic-bin-based null model analysis (iCAMP) was performed to explore community assembly mechanisms under different sediment carbon groups, by using the “iCAMP” package in R (Stegen et al., 2013; Ning et al., 2020).

## 3 Results

### 3.1 Physicochemical and nutrient characteristics of the reservoirs

**Figure 2:**
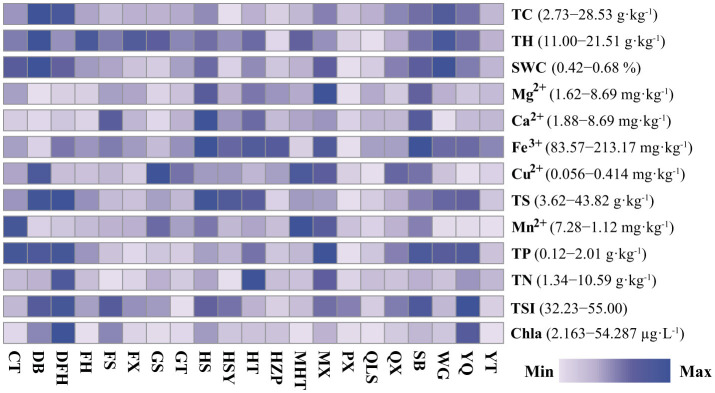
Heatmap displaying the changes in sediment and water environment parameters of the studied reservoirs in the Hanjiang river basin, southern China. The color scale for each environmental parameter varies between the minimum and maximum values. TC, sediment total carbon; SWC, sediment water content; TS, sediment total sulfur; TH, sediment total hydrogen; Ca^2+^, sediment calcium; Fe^3+^, sediment iron; Cu^2+^, sediment copper; Mn^2+^, sediment manganese; TP, sediment total phosphorus; TN, sediment total nitrogen; TSI, water trophic state index; Chla, water chlorophyll *a*. The locations and the full names of the studied reservoirs are given in Supplementary Table S2.

The 21 reservoirs exhibited a wide range of trophic state indices (TSI), spanning from oligotrophic (32–39) to mesotrophic (41–49) and eutrophic (above 50). Increasing eutrophication typically leads to higher total carbon (TC) content in sediments. Strong correlations were observed among water TSI, sediment TC, and sediment TP (Spearman's rank correlation, *r* > 0.405, *p* < 0.01), underscoring the significant relationship between reservoir eutrophication and sediment carbon accumulation (Supplementary Table S1). These findings suggest that eutrophication is a major driver of TC dynamics in sediments. Reservoir eutrophication led to increased total carbon (TC) in the sediments, with TC content ranging from 2.73 to 28.53 g·kg^−1^ (Figure 2). Sediment TC was positively correlated with water TSI, water chlorophyll *a* (Chla), sediment total phosphorus (TP), sediment total hydrogen (TH), sediment total sulfur (TS), and sediment water content (SWC) (Spearman's rank correlation, *r* > 0.405; *p* < 0.01; Supplementary Table S1). Significant correlations were also observed among sediment metal ions, including Mg^2+^, Ca^2+^, Fe^3+^, Cu^2+^, and Mn^2+^ (*p* < 0.05). Principal Component Analysis (PCA) reduced the environmental variables to two principal components (PC1 and PC2), which explained 32.82% and 22.75% of the total variation, respectively (Supplementary Figure S1). The strong correlations were found among water TSI, sediment TC, and sediment TP (Spearman's rank correlation, *r* > 0.405; *p* < 0.01), highlighting the significant relationships between reservoir eutrophication and sediment total carbon content (Supplementary Table S1).

### 3.2 Bacterial diversity and community composition in the reservoir sediments

A total of 3,387,770 high-quality bacterial sequences were obtained from the sediments of the 21 reservoirs. After quality filtering, 24,715 OTUs were retained, with 97% classified at the phylum level. A total of 73 phyla were identified, with the most abundant being Proteobacteria (21.56%), Chloroflexi (20.58%), Acidobacteria (9.30%), Bacteroidetes (7.86%), Verrucomicrobia (4.58%), Firmicutes (3.98%), Planctomycetes (3.64%), Actinobacteria (3.07%), and Cyanobacteria (0.99%). The remaining phyla accounted for 24.44% of the total bacterial community (Supplementary Figure S2). The relative abundances of Bacteroidetes, Firmicutes, and Planctomycetota were particularly high in the DuoBao (DB) and Dongfanghong (DFH) reservoirs, which had elevated nutrient levels in the sediments. In contrast, Chloroflexi, Acidobacteria, and Nitrospirota were more prevalent in the low-nutrient sediments of reservoirs such as Gangshan (GS), Yanqian (YQ), and Yitang (YT; Supplementary Figure S2). Notably, the Pingxi Reservoir (PX) exhibited the highest bacterial α-diversity, while the Heshanyan Reservoir (HSY) had the lowest (Supplementary Table S2).

### 3.3 Linking sediment bacterial community compositions with the environmental properties

**Figure 3:**
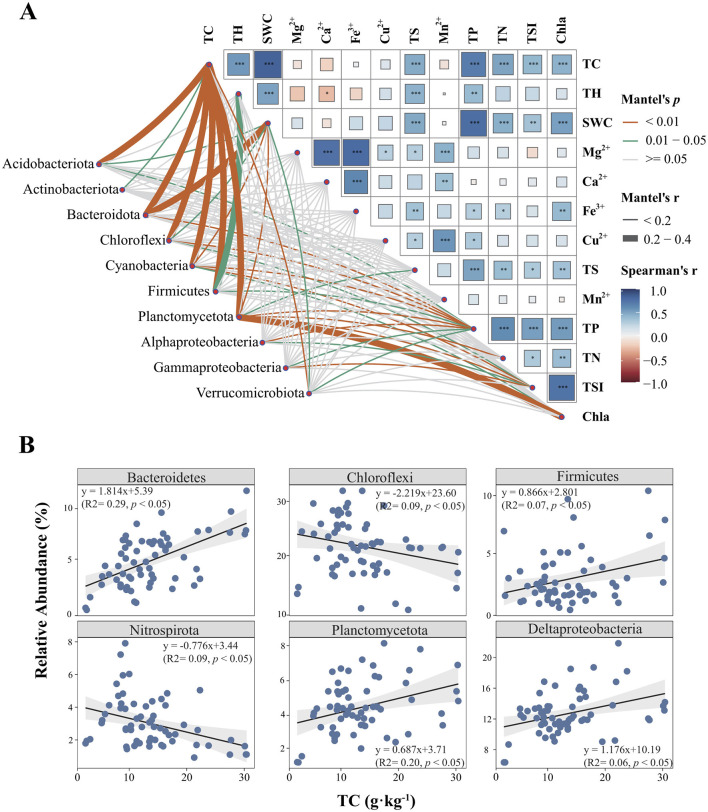
Relationships among environmental factors and their correlations with the relative abundance of the dominant (sub) phyla of bacterial communities **(A)**. The relative abundances of Bacteroidetes, Chloroflexi, Firmicutes, Nitrospirota, Planctomycetota, and Deltaproteobacteria along the TC gradient **(B)**. The relative abundance of Chloroflexi and Nitrospirota decreases linearly with increasing TC content, whereas Bacteroidetes, Firmicutes, Planctomycetota, and Deltaproteobacteria show positive correlations with TC content. TC, sediment total carbon; SWC, sediment water content; TS, sediment total sulfur; TH, sediment total hydrogen; Ca^2+^, sediment calcium; Fe^3+^, sediment iron; Cu^2+^, sediment copper; Mn^2+^, sediment manganese; TP, sediment total phosphorus; TN, sediment total nitrogen; TSI, water trophic state index; Chla, water chlorophyll *a*.

When relating the key sediment abiotic nutrient conditions (TP, TN, SWC, and TC), selected based on stepwise selection as the most influential variables, along with metals (Ca^2+^, Mn^2+^, Mg^2+^, Fe^3+^, and Cu^2+^) and geographical parameters, to bacterial community compositions (Supplementary Figure S3), we found that the variation in sediment bacterial compositions was uniquely explained by abiotic environmental variables (11.05%), metal compositions (1.98%), and geographical distances (1.39%) after accounting for shared effects among these factors. These results suggest that environmental factors related to sediment nutrient conditions exert a greater influence on bacterial community variation than metal composition and geographical distance. CCA-based ANOVA tests showed that the effects of these three sets of variables were all significant (*p* < 0.01). Among the measured environmental factors, TC showed the highest correlation with the bacterial community composition in reservoir sediments, followed by TP, TSI, and SWC as revealed by both canonical correspondence analyses (CCA, Supplementary Figure S3) and partial Mantel tests (Table 1). We further investigated the relationship between environmental factors and bacterial community composition. Significant correlations were found between multiple environmental factors (e.g., sediment TC, sediment TH, sediment TS, sediment TP, sediment TN, sediment SWC, water TSI, and water Chla) and the composition of bacterial phyla and subphyla (Figure 3A). Among them, the sediment TC showed a strong and significant correlation with the composition of several dominant bacterial phyla and subphyla, indicating that TC plays a key role in shaping the structure of sediment bacterial community. Additionally, we explored the relationships between TC and the relative abundance of the dominant phyla and subphyla (Figure 3A). Spearman's rank correlation analysis revealed a significant positive relationship between sediment TC and bacterial community composition, with sediment TC positively correlating with the relative abundance of Bacteroidetes, Firmicutes, Planctomycetota, and Deltaproteobacteria (r > 0.687, *p* < 0.01). Conversely, the relative abundance of Chloroflexi and Nitrospirota showed a significant negative correlation with TC (*r* < −0.776, *p* < 0.05; Figure 3B). At the sites with high TC, the major lineage or clades were affiliated from *Syntrophales, Clostridiaceae*, and *Bacteroidetes*_VadinHA17r, while *Anearolineaceae* dominated in low TC reservoirs (Supplementary Figure S4).

**Table 1:** Mantel test between transformed environmental variables and bacterial taxonomic composition (Bray-Curtis distance) and bacterial phylogenetic composition (UniFrac distance) based on Spearman's correlations.

**Factor**	**Taxonomic composition**		**Phylogenetic composition**	
	***R***	***p***	***R***	***p***
TC	0.357	0.001^***^	0.32	0.001^***^
TP	0.157	0.001^***^	0.129	0.001^***^
SWC	0.146	0.007^**^	0.123	0.012^*^
TSI	0.115	0.02^*^	0.147	0.03^*^
Chla	0.088	0.11	0.126	0.02^*^
TN	0.069	0.166	0.047	0.249
TH	0.056	0.198	0.012	0.424
TS	0.048	0.228	0.052	0.203
Cu^2+^	0.022	0.349	0.025	0.332
Mn^2+^	0.001	0.467	0.017	0.38
Mg^2+^	−0.058	0.805	−0.081	0.902
Ca^2+^	−0.021	0.594	−0.017	0.58
Fe^3+^	−0.019	0.6	−0.056	0.825

TC, sediment total carbon; SWC, sediment water content; TS, sediment total sulfur; TH, sediment total hydrogen; Ca^2+^, sediment calcium; Fe^3+^, sediment iron; Cu^2+^, sediment copper; Mn^2+^, sediment manganese; TP, sediment total phosphorus; TN, sediment total nitrogen; TSI, water trophic state index; Chla, water chlorophyll a.

^*^p < 0.05; ^**^p < 0.01; ^***^p < 0.001.

**Figure 4:**
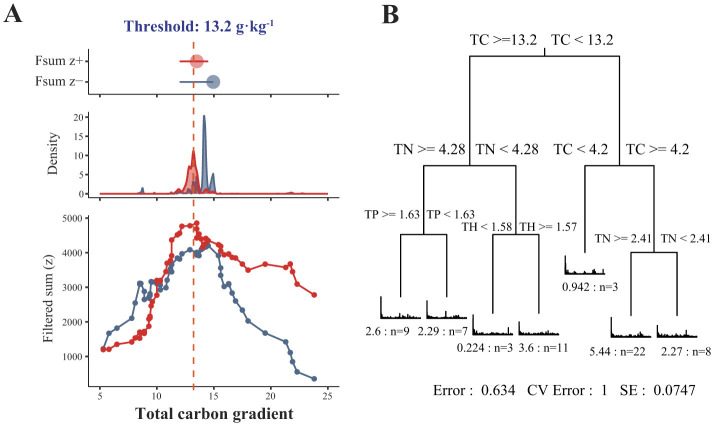
Threshold indicator taxon analysis of sediment bacterial communities in responses to TC content **(A)**. Red and blue symbols and areas indicate the magnitude of the summed z scores of increasing (z+) or decreasing (z–) taxa with an increasing TC content. The peak indicates the points where the sediment bacterial community structure gets large change along the total carbon gradient (the TC at the Z+ peak is approximately 13.2 g·kg^−1^). Multiple regression tree (MRT) with bacterial community composition as the response variable and environmental factors as the explanatory variables [**(B)**, *n* = 63].

We further evaluated the positive and negative niche thresholds for sediment bacteria in response to the changes of TC using TITAN. We observed that sediment bacteria had a TC threshold around 13.2 g·kg^−1^ (Figure 4A). This finding was further confirmed by the multiple regression tree (MRT) model with bacterial relative abundance as the response variable and environmental variables as the explanatory variables. Among the investigated environmental variables, the TC in the reservoir sediments caused divergences in the bacterial community structure, with a threshold of 13.2 g·kg^−1^ (Figure 4B).

### 3.4 Beta diversity patterns and community assembly processes of sediment bacteria in reservoirs of the Hanjiang river basin

**Figure 5:**
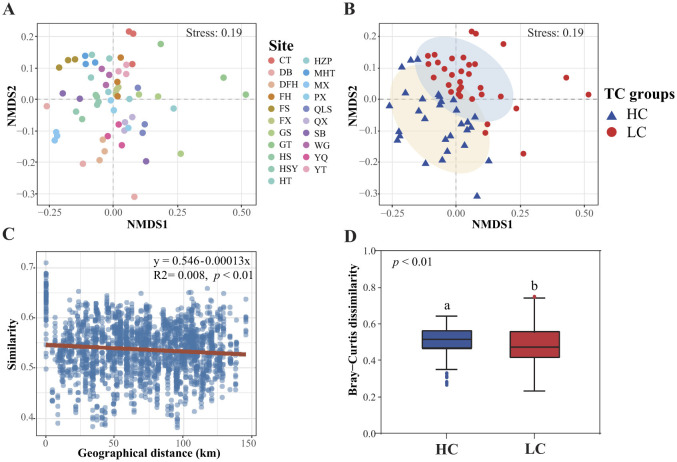
Non-metric Multidimensional Scaling (NMDS) shows sediment bacterial community structure arranging to sites **(A)** or to total carbon groups **(B)**. LC, low carbon content (< 13.2 g·kg^−1^) and HC, high carbon content (≥ 13.2 g·kg^−1^). Differences in bacterial community composition with geographical distance **(C)** as well as the beta diversity of bacterial communities in different total carbon groups **(D)**.

Non-metric Multidimensional Scaling (NMDS) analysis revealed that the spatial distributions of sediment bacterial communities in the reservoirs of the Hanjiang river basin did not follow clear geographical patterns (Figure 5A). Instead, they were grouped based on total carbon (TC) content into low-carbon (LC, TC < 13.2 g·kg^−1^) and high-carbon (HC, TC ≥ 13.2 g·kg^−1^) clusters (Figure 5B). PERMANOVA results confirmed significant differences in bacterial community structure between reservoirs with high and low TC content (*F* = 5.567, *p* < 0.001), indicating that eutrophication-driven carbon enrichment is a major factor shaping microbial biogeography (Figure 5B). The “distance-decay” relationship, which describes the correlation between bacterial community similarity and geographical distance, were notably weak (Mantel test, *R*^2^ = 0.008, *p* < 0.01; Figure 5C). However, beta diversity of sediment bacteria differed significantly between the LC and HC groups, with HC reservoir sediments exhibiting greater beta diversity (permutation ANOVA, *p* < 0.05) (Figure 5D).

### 3.5 Ecological assembly processes of sediment bacterial communities in reservoirs of the Hanjiang river basin

**Figure 6:**
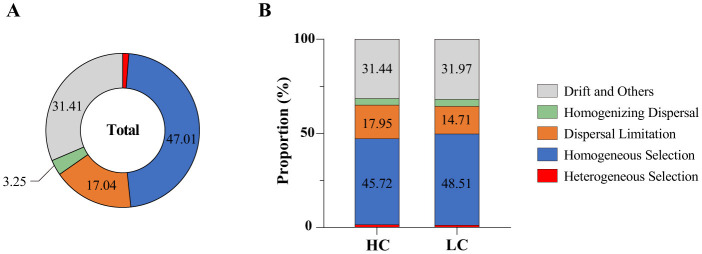
Relative importance of bacterial community assembly processes across all reservoirs **(A)** and within reservoirs with high (HC) and low (LC) total carbon content **(B)**.

The iCAMP analysis revealed that homogeneous selection played a dominant role in shaping bacterial community assembly in the reservoirs of the Hanjiang river basin. In high-carbon (HC) sediments, homogeneous selection accounted for 45.72% of the assembly processes, surpassing the contributions of drift (31.44%), and dispersal limitation (17.95%; Figure 6). Whereas, in low-carbon (LC) sediments, homogeneous selection remained the primary driver (48.51%), followed by drift (31.97%), and dispersal limitation (14.71%; Figure 6). Furthermore, the relative importance of homogeneous selection was lower in HC reservoirs compared to LC habitats (*t*-test, *p* < 0.05), highlighting the reduced influence of these processes in HC environments (Figures 6A, B).

### 3.6 Patterns of bacterial correlation in reservoirs of the Hanjiang river basin

**Figure 7:**
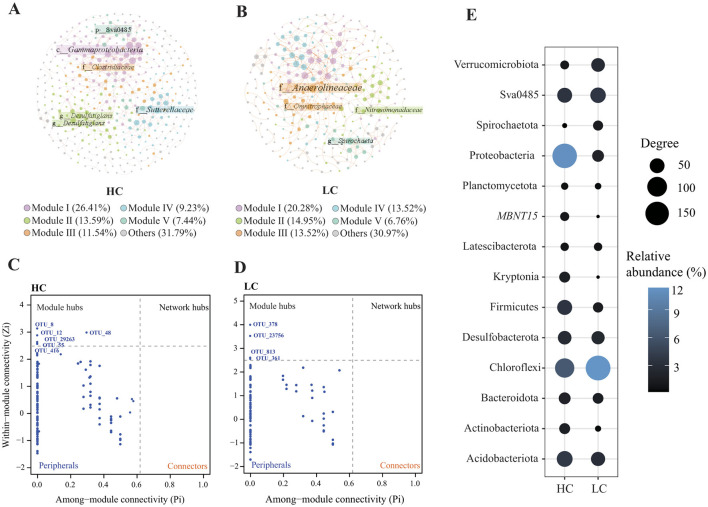
Correlation networks of the bacterial community in both high-carbon **(A)** and low-carbon **(B)** groups. The color of the nodes represents different modularity types. The within-module degree (Zi) and the participation coefficients (Pi) of each node can be used to identify their roles in high-carbon **(C)** and low-carbon **(D)** networks, respectively. The taxonomy of network nodes was showed at the phylum level **(E)**. The color represents the sum of phylum relative abundance, while the degree represents the sum of the phylum degrees in the network.

The HC network comprised 390 nodes and 505 edges, indicating a more complex bacterial community with greater taxonomic diversity and interaction potential, likely driven by the abundance of labile organic carbon substrates in eutrophic sediments (Zhou et al., 2020; Liu et al., 2021). In contrast, the LC network contained 281 nodes and 356 edges, reflecting a simpler community structure under oligotrophic conditions (Figure 7, Table 2). The higher number of nodes and edges in the HC network suggests that eutrophic conditions promote bacterial interactions, likely due to the enhanced availability of organic matter that supports diverse bacterial niches. Both networks exhibited small-world characteristics, as indicated by clustering coefficients and characteristic path lengths that significantly differed from corresponding random networks. The HC network displayed a higher average degree and clustering coefficient, reflecting more frequent and tighter associations among bacterial taxa. This suggests that bacterial communities in eutrophic sediments may exhibit stronger niche differentiation and interspecific competition, favoring functionally specialized bacterial groups that thrive in nutrient-rich environments (Table 2). Additionally, positive correlations (edges) outnumbered negative correlations in both networks (Figure 7), suggesting that bacterial taxa in both HC and LC sediments predominantly engage in cooperative interactions, such as cross-feeding or syntrophy. However, the proportion of negative correlations was notably higher in the HC network (12.08%) compared to the LC network (1.97%), indicating intensified niche segregation and competition among copiotrophic taxa in carbon-rich environments. This reflects the greater functional redundancy within eutrophic sediments, where multiple taxa compete for similar resources.

**Table 2:** Topological properties of sediment bacterial correlation networks in high-carbon (HC) and low-carbon (LC) reservoirs.

**Parameter**	**HC network**		**LC network**	
	**Observed**	**Random**	**Observed**	**Random**
No. of nodes	390	390	281	281
No. of edges	505	505	356	356
Positive proportion (%)	89.92		98.03	
Negative proportion (%)	12.08		1.97	
Clustering coefficient	0.322	0.007	0.299	0.016
Betweenness centrality	0.187	0.099	0.176	0.105
Average degree	2.590	2.590	2.534	2.534
Network centralization	0.187	0.069	0.176	0.112
Network density	0.007	0.006	0.009	0.010
Average path distance	7.631	5.148	9.264	5.2
Modularity	0.776	0.651	0.834	0.669
Network heterogeneity	0.902	0.604	0.827	0.656
Neighborhood connectivity	3.769	3.498	3.771	3.623
Harmonic geodesic distance	5.813	4.546	6.110	4.502

Furthermore, the modularity of the HC network (0.776) was lower than that of the LC network (0.834), suggesting that the bacterial communities in HC sediments are more interconnected, with less distinct ecological subgroups. This lower modularity may reflect a more dynamic bacterial community, with frequent shifts in dominant taxa in response to environmental fluctuations. Additionally, the robustness of the HC network (0.301) was lower than that of the LC network (0.358), indicating that the HC network is more susceptible to disruptions, likely due to the reliance of key taxa on eutrophic conditions. This is further supported by the vulnerability of the HC network (0.281), which was higher than that of the LC network (0.221), suggesting that bacterial communities in eutrophic sediments are more prone to structural collapse under perturbations, such as sudden changes in nutrient availability.

The Zi-Pi plots revealed that certain bacterial nodes functioned as keystone within the networks, acting as module hubs that connect nodes within a module (Figure 7). In the HC network, six keystone OTUs were identified, including two from Desulfobacterota (Desulfobacterota, OTU8 and OTU29263), one from *Clostridiaceae* (Firmicutes, OTU55), one from *Sutterellaceae* (Gammaproteobacteria, OTU416), and two from unclassified taxa. In contrast, the keystone OTUs in the LC network belonged to different taxa, including *Anaerolineaceae* (Chloroflexi, OTU278), *Spirochaeta* (Spirochaetota, OTU361), Candidatus *Omnitrophus* (Verrucomicrobiota, OTU813), and *Nitrosomonadaceae* (Gammaproteobacteria, OTU23756).

## 4 Discussion

Typically, geographically proximate reservoirs exhibit similar bacterial compositions due to shared geographic, hydrological, and climatic conditions, as well as common historical dispersal routes (Hanson et al., 2012; Liu et al., 2015; Viana et al., 2016; Gittins et al., 2023). However, the eutrophication-related nutrient changes of geographically proximate reservoirs may change bacterial biogeographic patterns at a regional scale (Tang et al., 2019; Amend et al., 2022). Our findings show that bacterial communities in geographically close reservoirs did not necessarily exhibit similar compositions. Instead, eutrophication-driven environmental selection, particularly related to total carbon levels, played a dominant role in shaping bacterial community structure. This influence was stronger than that of local environmental factors (e.g., temperature, salinity, and metal concentrations), random ecological events (e.g., drift), or geographic proximity (e.g., dispersal limitation or homogenizing dispersal).

At the regional scale, total carbon in sediments emerged as the key factor driving bacterial community patterns, reinforcing the idea that eutrophication-related environmental filtering plays a crucial role in microbial biogeography. This finding agrees with previous studies suggesting that bacterial community assembly in reservoirs is dominantly shaped by deterministic processes of some eutrophication-related environmental factors (Liu et al., 2019; Nyirabuhoro et al., 2020; Gittins et al., 2023). Total carbon in sediments may come mostly from phytoplankton, zooplankton, and bacteria (Nelson and Sommers, 1982; Yang et al., 2014). Our results showed that reservoirs with higher total carbon content also had higher trophic state index, chlorophyll *a*, total phosphorus, and total nitrogen, indicating that total carbon in reservoir sediments was significantly related to eutrophication (Li et al., 2020). The likely explanation is that reservoir eutrophication driven by anthropogenic activities resulted in the proliferation of algae, zooplankton, and heterotrophic microorganisms, leading to the storage of organic carbon on the sediment surface.

We further found that the total carbon content in reservoir sediments not only influenced the bacterial community compositions within individual reservoirs but also altered the regional biogeographic patterns of bacterial communities (Liu et al., 2019; Tang et al., 2019). The “distance-decay” relationship, which describes how bacterial community similarity declines with increasing geographic distance, is often used to assess ecosystem connectivity and microbial dispersal patterns. However, our study found that this relationship was weak, indicating that variations in total carbon, rather than geographic distance, were the primary factor influencing bacterial community composition across reservoirs. This suggests that eutrophication-driven TC enrichment disrupts the expected geographic structuring of microbial communities, reducing the strength of the typical “distance-decay” pattern. These differences in sediment total carbon extend local environmental factors, and shaped a broader-scale pattern of bacterial biogeography (Nyirabuhoro et al., 2020; Ren et al., 2023).

In our study, we found that total carbon (TC) content in reservoir sediments, driven by eutrophication, led to a distinct divergence in bacterial community structure, separating communities into high-carbon (HC) and low-carbon (LC) groups at a TC threshold of 13.2 g·kg^−1^. This threshold aligns with a significant shift in the trophic state index (TSI), transitioning from oligotrophic (32–39) to mesotrophic (41–49) and eutrophic conditions (above 50). Bacteroidetes and Firmicutes were significantly more abundant in HC sediments (TC > 13.2 g·kg^−1^; Wilcoxon test, *p* < 0.01), supporting their role in degrading labile organic matter under eutrophic conditions (Rasigraf et al., 2020; Chen et al., 2021). At the lineage or clade level, *Syntrophales* (Deltaproteobacteria), *Clostridiaceae* (Firmicutes), and VadinHA17 (Bacteroidetes) were particularly enriched in eutrophic, high-carbon environments (Tang et al., 2023; Liu et al., 2024). These taxa play complementary roles in the anaerobic carbon cycle of freshwater sediments, facilitating the efficient decomposition and recycling of organic matter (Coleman et al., 1993; Song et al., 2012). This process is a crucial step in the overall breakdown of organic material in HC sediments (Wan et al., 2017; Dong et al., 2022). Meanwhile, Chloroflexi, particularly *Anaerolineaceae*, exhibited a significantly higher relative abundance in LC sediments (TC < 13.2 g·kg^−1^; *p* < 0.05), indicating their adaptation to low-nutrient conditions and their ability to metabolize recalcitrant organic compounds (Hug et al., 2016). These findings align with previous studies demonstrating the functional divergence of microbial taxa in response to varying carbon availability (McNichol et al., 2024; Li et al., 2024). Meanwhile, certain taxa within Nitrospirota in LC sediments are capable of complete ammonia oxidation (comammox), directly converting ammonia to nitrate (Daims et al., 2015). This function is particularly important in nitrogen-limited environments, allowing Nitrospirota to thrive in nutrient-poor sediments and play a key role in sustaining the nitrogen cycle (Daims et al., 2015; Zhang et al., 2015). By integrating our findings with previous studies, we provide a broader ecological perspective on how sediment carbon and water TSI thresholds shape bacterial community compositions, contributing to a deeper understanding of bacterial responses to eutrophication in reservoir ecosystems (Filippini et al., 2019; Li et al., 2020, 2024). These insights underscore the importance of monitoring and managing sediment carbon levels to maintain balanced bacterial communities and ensure the health and stability of aquatic ecosystems (Lin and Han, 2001; Abell et al., 2008).

Both HC and LC reservoirs can exhibit regional heterogeneity in bacterial community compositions due to nutrient gradients, hydrodynamic variations, dispersal limitations, functional differentiation, and redox conditions (Huber et al., 2020; Li et al., 2020). However, in this study, we found that high carbon content in reservoir sediments increased regional heterogeneity in bacterial community compositions. This finding could be explained in several ways. First, that may be spatial heterogeneity in variations in carbon composition and quality across reservoirs. HC sediments provide diverse carbon substrates, including both labile and recalcitrant organic compounds, which promote niche differentiation among bacterial taxa (McNichol et al., 2024). For instance, copiotrophic bacteria (e.g., Bacteroidetes) dominate regions with labile organic matter. Oligotrophic bacteria (e.g., Chloroflexi) thrive in regions with more recalcitrant organic carbon (Li et al., 2024). Bacterial taxa specializing in different carbon substrates may dominate in localized reservoirs, leading to increased heterogeneity in community compositions at a regional scale (Landa et al., 2016). Second, the accumulation of total carbon in eutrophic sediments facilitates the formation of distinct redox gradients and microhabitats, supporting a broader range of bacterial taxa with varying oxygen and nutrient requirements (Li et al., 2020, 2024). For example, sulfate-reducing Deltaproteobacteria were significantly more abundant in HC reservoirs (*p* < 0.05), likely benefiting from localized anoxic conditions that develop as organic matter decomposes (Rasigraf et al., 2020). This spatial heterogeneity within sediments may explain the increased microbial community divergence observed in HC reservoirs at the regional scale. Third, that could be attributed to community assembly mechanisms which amplify regional variation (Viana et al., 2016; Huber et al., 2020). Regional-scale dispersal barriers (e.g., physical separation of reservoirs or isolated sediment patches) interact with local HC conditions, preventing homogenization of bacterial communities across reservoirs at a regional scale (Chase and Ryberg, 2004; Chen et al., 2019; Huber et al., 2020). Moreover, HC might diminish the environmental filtering effects of local conditions (e.g., TSI, TP, Chla, and SWC), leading to reduced homogeneity among bacterial taxa across different reservoirs (Zeng et al., 2019; Zuo et al., 2024). Our findings revealed that reservoirs with HC exhibited a reduced influence of homogeneous selection on sediment bacterial community assembly compared to LC habitats. Collectively, these three ways might lead to distinct bacterial assemblages in individual reservoirs, increasing the heterogeneity of bacterial community compositions at the regional scale.

Bacterial correlation networks offer valuable insights into how bacterial taxa coexist through niche differentiation or resource partitioning (Faust and Raes, 2012; He et al., 2021). In this study, we observed that the sediment bacterial correlation network in eutrophic reservoirs with high total carbon levels was more intricate, featuring a greater number of nodes and edges, compared to networks in low-carbon reservoirs. This observation aligns with ecological theories suggesting that resource enrichment, such as carbon input in eutrophic systems, can increase bacterial interaction complexity (Hernandez et al., 2021; Guo et al., 2023). However, it also highlights potential risks, such as reduced network stability or dominance of opportunistic taxa in highly nutrient-enriched environments (Goyal and Maslov, 2018; Mikhailov et al., 2019; Zeng et al., 2019). The HC network exhibits lower robustness and higher vulnerability, indicating that although eutrophication may promote complex interactions, such as mutualism, commensalism, or competition, it renders the bacterial network more fragile and prone to collapse under environmental perturbations (Widder et al., 2014; Fang et al., 2023). In our study, we found that the number of positive correlations (edges) exceeded that of negative correlations in both LC and HC bacterial networks; however, the proportion of negative correlations was higher in the HC network compared to the LC network. This suggests that mutualism or commensalism dominated in bacterial communities of both LC and HC reservoirs. But bacterial communities in HC reservoirs exhibited stronger competition and greater niche segregation compared to those in LC reservoirs. In eutrophic conditions, bacteria may adapt to utilize different niches or partition resources to coexist (Mougi and Kondoh, 2012; Morriën et al., 2017). This niche segregation fostered the coexistence of more taxa, further increasing network complexity (Hernandez et al., 2021). In addition, we found that sediment bacterial correlation networks in high total carbon reservoirs (eutrophic conditions) exhibited higher clustering characteristics, as evidenced by a greater average clustering coefficient, shorter harmonic geodesic distances, and lower modularity, compared to networks in low carbon reservoirs. Higher clustering networks in eutrophic systems might be more sensitive to environmental disturbances, as disruptions in key nodes or interactions can ripple through the network (Goyal and Maslov, 2018; Banerjee et al., 2019).

Keystone nodes, identified through network centrality metrics, play a disproportionately significant role in maintaining the stability and functionality of community networks and in preventing assemblage fragmentation (Faust and Raes, 2012). The keystone OTUs in high-carbon sediment networks were found mainly affiliated with Desulfobacterota*, Clostridiaceae*, and *Sutterellaceae*. These taxa act as metabolic hubs in high-carbon sediments (Wang et al., 2021; Ren et al., 2022; Yu et al., 2022; Yadav et al., 2024). They also produce carbon metabolites that are vital for supporting syntrophic interactions involved in methanogenesis, denitrification, and sulfate reduction (Zhang et al., 2010; Wang et al., 2022). In low-carbon sediments, the keystone OTUs belonged to *Anaerolineaceae* (related to OTU278) and *Spirochaeta* (related to OTU361), which sustained carbon metabolisms in sediments by breaking down recalcitrant organic carbon efficiently (Xie et al., 2021). This process provides them with energy to fix carbon dioxide via the Calvin cycle, contributing to primary production and supporting bacterial food webs in environments with low carbon availability (Dong et al., 2024). These findings underscore the significant influence of eutrophication-driven changes in total carbon on sediment bacterial correlation patterns, highlighting the interplay between external environmental changes and sediment bacterial interactions in shaping niche differentiation and resource partitioning within reservoirs.

## 5 Conclusion

Our results revealed that eutrophication-driven increases in total carbon (TC) profoundly altered the regional biogeographic patterns of sediment bacterial communities. These changes weakened the typical “distance-decay” relationships, where bacterial community similarity decreases with geographical distance. When TC concentrations exceeded a threshold of 13.2 g·kg^−1^, there were pronounced shifts in bacterial community structure. Moreover, higher TC content was linked to greater regional heterogeneity in bacterial community composition. Reservoirs with high TC levels exhibited more intricate microbial interaction networks, characterized by stronger niche segregation and higher competition compared to low TC networks.

These findings highlight the critical role of sediment TC in shaping microbial biogeography at a regional scale. More importantly, they provide valuable insights for the management of water quality and microbial community restoration in eutrophic freshwater ecosystems, particularly in subtropical reservoirs. Given that high TC levels can increase microbial network complexity but also reduce stability, targeted strategies should focus on maintaining a balance between nutrient enrichment and microbial resilience. Management efforts, such as reducing external carbon inputs from agricultural runoff and wastewater discharge, could help mitigate excessive network fragility and prevent functional losses in sediment microbial communities. Additionally, restoration approaches that promote keystone microbial taxa involved in carbon and nitrogen cycling could enhance ecosystem stability and improve water quality. By elucidating the ecological consequences of TC-driven eutrophication, our study offers a scientific basis for developing effective mitigation strategies that minimize anthropogenic impacts on freshwater ecosystems. Future research should further explore how dynamic nutrient management and microbial community interventions can enhance ecosystem resilience in the face of ongoing environmental change.

## Data Availability

The sequencing data obtained were deposited into the Sequence Read Archive (SRA) of the National Center for Biotechnology Information (https://www.ncbi.nlm.nih.gov/subs/sra) database under accession number PRJNA1108051 (TaxID:749907). Other environmental data will be made available on request.

## References

[B1] Abell R. Thieme M. L. Revenga C. Bryer M. Kottelat M. Bogutskaya N.. (2008). Freshwater ecoregions of the world: a new map of biogeographic units for freshwater biodiversity conservation. Bioscience 58, 403–414. 10.1641/B580507

[B2] Amend A. S. Swift S. O. I. Darcy J. L. Belcaid M. Nelson C. E. Buchanan J.. (2022). A ridge-to-reef ecosystem microbial census reveals environmental reservoirs for animal and plant microbiomes. Proc. Natl. Acad. Sci. U.S.A. 119:e2204146119. 10.1073/pnas.2204146119 35960845 PMC9388140

[B3] Anderson M. J. (2014). “Permutational multivariate analysis of variance (PERMANOVA),” in Wiley StatsRef: Statistics Reference Online (Hoboken, NJ: John Wiley & Sons, Ltd).

[B4] Banerjee S. Walder F. Büchi L. Meyer M. Held A. Y. Gattinger A.. (2019). Agricultural intensification reduces microbial network complexity and the abundance of keystone taxa in roots. ISME J. 13, 1722–1736. 10.1038/s41396-019-0383-2 30850707 PMC6591126

[B5] Bolger A. M. Lohse M. Usadel B. (2014). Trimmomatic: a flexible trimmer for Illumina sequence data. Bioinformatics 30, 2114–2120. 10.1093/bioinformatics/btu170 24695404 PMC4103590

[B6] Buchan A. LeCleir G. R. Gulvik C. A. González J. M. (2014). Master recyclers: features and functions of bacteria associated with phytoplankton blooms. Nat. Rev. Microbiol. 12, 686–698. 10.1038/nrmicro3326 25134618

[B7] Carlson R. E. (1977). A trophic state index for lakes. Limnol. Oceanogr. 22, 361–369. 10.4319/lo.1977.22.2.0361

[B8] Chase J. M. Ryberg W. A. (2004). Connectivity, scale-dependence, and the productivity–diversity relationship. Ecol. Lett. 7, 676–683. 10.1111/j.1461-0248.2004.00622.x

[B9] Chen W. Ren K. Isabwe A. Chen H. Liu M. Yang J. (2019). Correction to: stochastic processes shape microeukaryotic community assembly in a subtropical river across wet and dry seasons. Microbiome 7:148. 10.1186/s40168-019-0763-x 31727140 PMC6857158

[B10] Chen Y. J. Leung P. M. Wood J. L. Bay S. K. Hugenholtz P. Kessler A. J.. (2021). Metabolic flexibility allows bacterial habitat generalists to become dominant in a frequently disturbed ecosystem. ISME J. 15, 2986–3004. 10.1038/s41396-021-00988-w 33941890 PMC8443593

[B11] Coleman M. L. Hedrick D. B. Lovley D. R. White D. C. Pye K. (1993). Reduction of Fe(III) in sediments by sulphate-reducing bacteria. Nature 361, 436–438. 10.1038/361436a0

[B12] Daims H. Lebedeva E. V. Pjevac P. Han P. Herbold C. Albertsen M.. (2015). Complete nitrification by *Nitrospira* bacteria. Nature 528, 504–509. 10.1038/nature16461 26610024 PMC5152751

[B13] De Borba B. M. Jack R. F. Rohrer J. S. Wirt J. Wang D. (2014). Simultaneous determination of total nitrogen and total phosphorus in environmental waters using alkaline persulfate digestion and ion chromatography. J. Chromatogr. A. 1369, 131–137. 10.1016/j.chroma.2014.10.027 25441080

[B14] Dong L. Liu Z. Xin Z. Song C. Bai X. Li J.. (2024). Runoff variation alters estuarine sediment microbiome and nitrogen removal processes by affecting salinity. Sci. Total Environ. 955:176880. 10.1016/j.scitotenv.2024.176880 39419209

[B15] Dong X. Zhang C. Peng Y. Zhang H.-X. Shi L-D. Wei G.. (2022). Phylogenetically and catabolically diverse diazotrophs reside in deep-sea cold seep sediments. Nat. Commun. 13:4885. 10.1038/s41467-022-32503-w 35985998 PMC9391474

[B16] D'Souza G. Shitut S. Preussger D. Yousif G. Waschina S. Kost C.. (2018). Ecology and evolution of metabolic cross-feeding interactions in bacteria. Nat. Prod. Rep. 35, 455–488. 10.1039/C8NP00009C 29799048

[B17] Edgar R. C. (2013). UPARSE: highly accurate OTU sequences from microbial amplicon reads. Nat. Methods 10, 996–998. 10.1038/nmeth.2604 23955772

[B18] Fang W. Fan T. Xu L. Wang S. Wang X. Lu A.. (2023). Seasonal succession of microbial community co-occurrence patterns and community assembly mechanism in coal mining subsidence lakes. Front. Microbiol. 14:1098236. 10.3389/fmicb.2023.1098236 36819062 PMC9936157

[B19] Faust K. Raes J. (2012). Microbial interactions: from networks to models. Nat. Rev. Microbiol. 10, 538–550. 10.1038/nrmicro2832 22796884

[B20] Figary S. DeWitt P. Detenbeck N. (2022). pTITAN2: permutation of treatment labels and threshold indicator taxa ANalysis. F1000Research 11:267. 10.12688/f1000research.83714.1

[B21] Filippini G. Bugnot A. B. Johnston E. L. Ruszczyk J. Potts J. Scanes P.. (2019). (2019). Sediment bacterial communities associated with environmental factors in Intermittently Closed and Open Lakes and Lagoons (ICOLLs). *Sci. Total Environ*. 693:133462. 10.1016/j.scitotenv.2019.07.268 31374508

[B22] Gittins D. A. Bhatnagar S. Hubert C. R. J. (2023). Environmental selection and biogeography shape the microbiome of subsurface petroleum reservoirs. mSystems 8:e0088422. 10.1128/msystems.00884-22 36786580 PMC10134868

[B23] Goyal A. Maslov S. (2018). Diversity, stability, and reproducibility in stochastically assembled microbial ecosystems. Phys. Rev. Lett. 120:158102. 10.1103/PhysRevLett.120.158102 29756882

[B24] Greenberg A. E. Trussell R. R. Clesceri L. S. American Public Health Association American Water Work Association. (1988). Standard Methods for the Examination of Water and Wastewate, 16th Edn. Washington, DC: American Public Health Association.

[B25] Guo Z. Li Y. Shao M. Sun T. Lin M. Zhang T.. (2023). Succession and environmental response of sediment bacterial communities in the Liao river estuary at the centenary scale. Mar. Environ. Res. 188:105980. 10.1016/j.marenvres.2023.105980 37141709

[B26] Han B. P. Dumont H. J. (2011). Reservoirs of Guangdong province, South China: an increasing threat of eutrophication. Oecologia Australis 15, 643–654. 10.4257/oeco.2011.1503.15

[B27] Hanson C. A. Fuhrman J. A. Horner-Devine M. C. Martiny J. B. (2012). Beyond biogeographic patterns: processes shaping the microbial landscape. Nat. Rev. Microbiol. 10, 497–506. 10.1038/nrmicro2795 22580365

[B28] He Q. Wang S. Hou W. Feng K. Li F. Hai W.. (2021). Temperature and microbial interactions drive the deterministic assembly processes in sediments of hot springs. Sci. Total Environ. 772:145465. 10.1016/j.scitotenv.2021.145465 33571767

[B29] Hernandez D. J. David A. S. Menges E. S. Searcy C. A. Afkhami M. E. (2021). Environmental stress destabilizes microbial networks. ISME J. 15, 1722–1734. 10.1038/s41396-020-00882-x 33452480 PMC8163744

[B30] Huber P. Metz S. Unrein F. Mayora G. Sarmento H. Devercelli M.. (2020). Environmental heterogeneity determines the ecological processes that govern bacterial metacommunity assembly in a floodplain river system. ISME J. 14, 2951–2966. 10.1038/s41396-020-0723-2 32719401 PMC7784992

[B31] Hug L. A. Thomas B. C. Sharon I. Brown C. T. Sharma R. Hettich R. L.. (2016). Critical biogeochemical functions in the subsurface are associated with bacteria from new phyla and little studied lineages. Environ. Microbiol. 18, 159–173. 10.1111/1462-2920.12930 26033198

[B32] Landa M. Blain S. Christaki U. Monchy S. Obernosterer I. (2016). Shifts in bacterial community composition associated with increased carbon cycling in a mosaic of phytoplankton blooms. ISME J. 10, 39–50. 10.1038/ismej.2015.105 26196334 PMC4681851

[B33] Layeghifard M. Hwang D. M. Guttman D. S. (2017). Disentangling interactions in the microbiome: a network perspective. Trends Microbiol. 25, 217–228. 10.1016/j.tim.2016.11.008 27916383 PMC7172547

[B34] Li C. P. Li Y. P. Huo Q. Q. Xiao W. Duan C. Q. Wang Y. X.. (2020). Comparison of prokaryotic communities associated with different TOC concentrations in Dianchi lake. Water 12:2557. 10.3390/w12092557

[B35] Li Y. Chen Z. Zhang N. Zheng H. Miao Y. Li J.. (2024). Decreased microbial phylogenetic diversity and community stability due to less bioavailable carbon and greater oxygen supply in Mollisols along a cultivation chronosequence. Soil Tillage Res. 238:106005. 10.1016/j.still.2024.106005

[B36] Lin Q. Han B. (2001). Reservior limnology and its application in water quality management an overview. Acta Ecol. Sin. 21, 1034–1040.

[B37] Liu C. Wang X. Li X. Yang Z. Dang K. Gong X.. (2024). Effects of intercropping on rhizosphere microbial community structure and nutrient limitation in proso millet/mung bean intercropping system. Eur. J. Soil Biol. 122:103646. 10.1016/j.ejsobi.2024.103646

[B38] Liu L. Chen H. Liu M. Yang J. R. Xiao P. Wilkinson D. M.. (2019). Response of the eukaryotic plankton community to the cyanobacterial biomass cycle over 6 years in two subtropical reservoirs. ISME J. 13, 2196–2208. 10.1038/s41396-019-0417-9 31053831 PMC6776060

[B39] Liu L. Yang J. Yu Z. Wilkinson D. M. (2015). The biogeography of abundant and rare bacterioplankton in the lakes and reservoirs of China. ISME J. 9, 2068–2077. 10.1038/ismej.2015.29 25748371 PMC4542038

[B40] Liu L. L. Sun F. F. Zhao H. B. Mi H. S. He S. Q. Chen Y.. (2021). Compositional changes of sedimentary microbes in the Yangtze river estuary and their roles in the biochemical cycle. Sci. Total Environ. 760:143383. 10.1016/j.scitotenv.2020.143383 33189382

[B41] McNichol S. M. Sanchez-Quete F. Loeb S. K. Teske A. P. Shah Walter S. R. Mahmoudi N.. (2024). Dynamics of carbon substrate competition among heterotrophic microorganisms. ISME J. 18:wrae018. 10.1093/ismejo/wrae018 38366177 PMC10942773

[B42] Meziti A. Tsementzi D. Kormas A. K. Karayanni H. Konstantinidis K. T. (2016). Anthropogenic effects on bacterial diversity and function along a river-to-estuary gradient in Northwest Greece revealed by metagenomics. Environ. Microbiol. 18, 4640–4652. 10.1111/1462-2920.13303 27001690

[B43] Mikhailov I. S. Zakharova Y. R. Bukin Y. S. Galachyants Y. P. Petrova D. P. Sakirko M. V.. (2019). Co-occurrence networks among bacteria and microbial eukaryotes of lake Baikal during a spring phytoplankton bloom. Microb. Ecol. 77:558. 10.1007/s00248-018-1307-9 30610256

[B44] Morriën E. Hannula S. E. Snoek L. B. Helmsing N. R. Zweers H. Hollander D.. (2017). Soil networks become more connected and take up more carbon as nature restoration progresses. Nat. Commun. 8:14349. 10.1038/ncomms14349 28176768 PMC5309817

[B45] Mougi A. Kondoh M. (2012). Diversity of interaction types and ecological community stability. Science 337, 349–351. 10.1126/science.1220529 22822151

[B46] Nelson D. W. Sommers L. E. (1982). “Total carbon, organic carbon, and organic matter,” in Methods of Soil Analysis: Part 2 Chemical and Microbiological Properties, eds. D. L. Sparks, A. L. Page, P. A. Helmke, R. H. Loeppert, P. N. Soltanpour, M. A. Tabatabai, et al., (Madison, WI: SSSA and AAA), 539–579.

[B47] Newton R. J. Jones S. E. Eiler A. McMahon K. D. Bertilsson S. (2011). A guide to the natural history of freshwater lake bacteria. Microbiol. Mol. Biol. Rev. 75, 14–49. 10.1128/MMBR.00028-10 21372319 PMC3063352

[B48] Nilsson C. Berggren K. (2000). Alterations of riparian ecosystems caused by river regulation: dam operations have caused global-scale ecological changes in riparian ecosystems. How to protect river environments and human needs of rivers remains one of the most important questions of our time. Bioscience 50, 783–792. 10.1641/0006-3568(2000)050[0783:AORECB]2.0.CO;2

[B49] Ning D. Yuan M. Wu L. Zhang Y. Guo X. Zhou X.. (2020). A quantitative framework reveals ecological drivers of grassland microbial community assembly in response to warming. Nat. Commun. 11:4717. 10.1038/s41467-020-18560-z 32948774 PMC7501310

[B50] Nyirabuhoro P. Liu M. Xiao P. Liu L. Yu Z. Wang L.. (2020). Seasonal variability of conditionally rare taxa in the water column bacterioplankton community of subtropical reservoirs in China. Microb. Ecol. 80, 14–26. 10.1007/s00248-019-01458-9 31836929

[B51] Parks D. H. Beiko R. G. (2010). Identifying biologically relevant differences between metagenomic communities. Bioinformatics 26, 715–721. 10.1093/bioinformatics/btq041 20130030

[B52] Pearson D. E. Ortega Y. K. Eren Ö. Hierro J. L. (2018). Community assembly theory as a framework for biological invasions. Trends Ecol. Evol. 33, 313–325. 10.1016/j.tree.2018.03.002 29605085

[B53] Pinto I. Rodrigues S. Lage O. Antunes S. (2021). Assessment of water quality in Aguieira reservoir: ecotoxicological tools in addition to the water framework directive. Ecotoxicol. Environ. Saf. 208:111583. 10.1016/j.ecoenv.2020.111583 33396106

[B54] PrŽulj N. Malod-Dognin N. (2016). Network analytics in the age of big data. Science 353, 123–124. 10.1126/science.aah3449 27387938

[B55] Quast C. Pruesse E. Yilmaz P. Gerken J. Schweer T. Yarza P.. (2012). The SILVA ribosomal RNA gene database project: improved data processing and web-based tools. Nucleic Acids Res. 41, D590–D596. 10.1093/nar/gks1219 23193283 PMC3531112

[B56] Rasigraf O. van Helmond N. A. G. M. Frank J. Lenstra W. K. Egger M. Slomp C. P.. (2020). Microbial community composition and functional potential in Bothnian sea sediments is linked to Fe and S dynamics and the quality of organic matter. Limnol. Oceanogr. 65, S113–S133. 10.1002/lno.11371

[B57] Reitter C. Petzoldt H. Korth A. Schwab F. Stange C. Hambsch B.. (2021). Seasonal dynamics in the number and composition of coliform bacteria in drinking water reservoirs. Sci. Total Environ. 787:147539. 10.1016/j.scitotenv.2021.147539

[B58] Ren L. Song X. Wu C. Li G. Zhang X. Xia X.. (2023). Biogeographical and biodiversity patterns of marine planktonic bacteria spanning from the South China sea across the Gulf of Bengal to the northern Arabian Sea. Microbiol. Spectr. 11, e00398–e00323. 10.1128/spectrum.00398-23 37098981 PMC10269852

[B59] Ren Z. Cao S. Chen T. Zhang C. Yu J. (2022). Bacterial functional redundancy and carbon metabolism potentials in soil, sediment, and water of thermokarst landscapes across the Qinghai-Tibet Plateau: implications for the fate of permafrost carbon. Sci. Total Environ. 852:158340. 10.1016/j.scitotenv.2022.158340 36041614

[B60] Säwström C. Serrano O. Rozaimi M. Lavery P. S. (2016). Utilization of carbon substrates by heterotrophic bacteria through vertical sediment profiles in coastal and estuarine seagrass meadows. Environ. Microbiol. Rep. 8, 582–589. 10.1111/1758-2229.12406 27188411

[B61] Sinsabaugh R. L. Belnap J. Findlay S. G. Shah J. J. F. Hill B. H. Kuehn K. A.. (2014). Extracellular enzyme kinetics scale with resource availability. Biogeochemistry 121, 287–304. 10.1007/s10533-014-0030-y20503877

[B62] Smith V. H. (2003). Eutrophication of freshwater and coastal marine ecosystems a global problem. *Environ. Sci. Pollut. Res*. Int. 10, 126–139. 10.1065/espr2002.12.142 12729046

[B63] Smith V. H. Schindler D. W. (2009). Eutrophication science: where do we go from here? Trends Ecol. Evol. 24, 201–207. 10.1016/j.tree.2008.11.009 19246117

[B64] Song H. Li Z. Du B. Wang G. Ding Y. (2012). Bacterial communities in sediments of the shallow Lake Dongping in China. J. Appl. Microbiol. 112, 79–89. 10.1111/j.1365-2672.2011.05187.x 22044641

[B65] Stegen J. C. Lin X. Konopka A. E. Fredrickson J. K. (2012). Stochastic and deterministic assembly processes in subsurface microbial communities. ISME J. 6, 1653–1664. 10.1038/ismej.2012.22 22456445 PMC3498916

[B66] Stegen J. C. Lin X. J. Fredrickson J. K. Chen X. Y. Kennedy D. W. Murray C. J.. (2013). Quantifying community assembly processes and identifying features that impose them. ISME J. 7, 2069–2079. 10.1038/ismej.2013.93 23739053 PMC3806266

[B67] Sun M. Y. Dafforn K. A. Brown M. V. Johnston E. L. (2012). Bacterial communities are sensitive indicators of contaminant stress. Mar. Pollut. Bull. 64, 1029–1038. 10.1016/j.marpolbul.2012.01.035 22385752

[B68] Tang F. Zhang H. Cheng H. Wang Y. Liu Q. Zhao C.. (2023). New insights of crude oil biodegradation construction by microbial consortium B10: responded substrates, genomics, biodegradation mechanism and pathways. Chem. Eng. J. 478:147143. 10.1016/j.cej.2023.147143

[B69] Tang Q. Peng L. Yang Y. Lin Q. Qian S. S. Han B. P.. (2019). Total phosphorus-precipitation and chlorophyll *a*-phosphorus relationships of lakes and reservoirs mediated by soil iron at regional scale. Water Res. 154, 136–143. 10.1016/j.watres.2019.01.038 30782555

[B70] Thomas C. Francke A. Vogel H. Wagner B. Ariztegui D. (2020). Weak influence of paleoenvironmental conditions on the subsurface biosphere of Lake Ohrid over the last 515 ka. Microorganisms 8:1736. 10.3390/microorganisms8111736 33167482 PMC7716225

[B71] Viana D. S. Figuerola J. Schwenk K. Manca M. Hobæk A. Mjelde M.. (2016). Assembly mechanisms determining high species turnover in aquatic communities over regional and continental scales. Ecography 39, 281–288. 10.1111/ecog.01231

[B72] Wallenius A. J. Martins P. D. Slomp C. P. Jetten M. S. M. (2021). Anthropogenic and environmental constraints on the microbial methane cycle in coastal sediments. Front. Microbiol. 12:631621. 10.3389/fmicb.2021.631621 33679659 PMC7935538

[B73] Wan Y. Ruan X. Zhang Y. Li R. (2017). Illumina sequencing-based analysis of sediment bacteria community in different trophic status freshwater lakes. MicrobiologyOpen 6:e00450. 10.1002/mbo3.450 28173613 PMC5552931

[B74] Wang C. Pan X. Yu W. Ye X. Erdenebileg E. Wang C.. (2023). Aridity and decreasing soil heterogeneity reduce microbial network complexity and stability in the semi-arid grasslands. Ecol. Indicators. 151:110342. 10.1016/j.ecolind.2023.110342

[B75] Wang H. Gu Y. Zhou W. Zhao D. Qiao Z. Zheng J.. (2021). Adaptability of a caproate-producing bacterium contributes to its dominance in an anaerobic fermentation system. Appl. Environ. Microbiol. 87, e01203–e01221. 10.1128/AEM.01203-21 34378978 PMC8478455

[B76] Wang J. Wei Z-P. Chu Y. X. Tian G. He R. (2022). Eutrophic levels and algae growth increase emissions of methane and volatile sulfur compounds from lakes. Environ. Pollut. 306:119435. 10.1016/j.envpol.2022.119435 35550131

[B77] Wang W. Yang Y. Zhou Y. Zhang S. Wang X. Yang Z.. (2020). Impact of anthropogenic activities on the sediment microbial communities of Baiyangdian shallow lake. Int. J. Sediment Res. 35, 180–192. 10.1016/j.ijsrc.2019.10.006

[B78] Wang Y-C. Lv Y-H. Wang C. Deng Y. Lin Y-T. Jiang G-Y.. (2024). Stochastic processes shape microbial community assembly in biofilters: hidden role of rare taxa. Bioresour. Technol. 402:130838. 10.1016/j.biortech.2024.130838 38740312

[B79] Widder S. Besemer K. Singer G. A. Ceola S. Bertuzzo E. Quince C.. (2014). Fluvial network organization imprints on microbial co-occurrence networks. Proc. Natl. Acad. Sci. U.S.A. 111, 12799–12804. 10.1073/pnas.1411723111 25136087 PMC4156742

[B80] Xie E. Zhao X. Li K. Zhang P. Zhou X. Zhao X.. (2021). Microbial community structure in the river sediments from upstream of Guanting reservoir: potential impacts of reclaimed water recharge. Sci. Total Environ. 766:142609. 10.1016/j.scitotenv.2020.142609 33069478

[B81] Xu X. Wang N. Lipson D. Sinsabaugh R. Schimel J. He L.. (2020). Microbial macroecology: in search of mechanisms governing microbial biogeographic patterns. Global Ecol. Biogeogr. 29, 1870–1886. 10.1111/geb.13162

[B82] Yadav S. Koenen M. Bale N. J. Reitsma W. Engelmann J. C. Stefanova K.. (2024). Organic matter degradation in the deep, sulfidic waters of the Black sea: insights into the ecophysiology of novel anaerobic bacteria. Microbiome 12:98. 10.1186/s40168-024-01816-x 38797849 PMC11129491

[B83] Yang J. Yu X. Liu L. Zhang W. Guo P. (2012). Algae community and trophic state of subtropical reservoirs in southeast Fujian, China. Environ. Sci. Pollut. Res. 19, 1432–1442. 10.1007/s11356-011-0683-1 22743992

[B84] Yang N. Zhang C. Wang L. Li Y. Zhang W. Niu L.. (2021). Nitrogen cycling processes and the role of multi-trophic microbiota in dam-induced river-reservoir systems. Water Res. 206:117730. 10.1016/j.watres.2021.117730 34619413

[B85] Yang Y. Liu Q. Hu Z. Zhang Y. Gao Y. (2014). Spatial distribution of sediment carbon, nitrogen and phosphorus and pollution evaluation of sediment in Taihu lake basin. Acta Sci. Circumst. 34, 3057–3064. 10.13671/j.hjkxxb.2014.0710

[B86] Yu B. Zeng Q. Li J. Li J. Tan X. Gao X.. (2023). Vertical variation in prokaryotic community composition and co-occurrence patterns in sediments of the three Gorges reservoir, China. Environ. Res. 237:116927. 10.1016/j.envres.2023.116927 37604225

[B87] Yu H. Zhong Q. Peng Y. Zheng X. Xiao F. Wu B.. (2022). Environmental filtering by pH and salinity jointly drives prokaryotic community assembly in coastal wetland sediments. Front. Mar. Sci. 8:792294. 10.3389/fmars.2021.792294

[B88] Yuan M. M. Guo X. Wu L. Zhang Y. Xiao N. Ning D.. (2021). Climate warming enhances microbial network complexity and stability. Nat. Clim. Change 11, 343–348. 10.1038/s41558-021-00989-9

[B89] Zarri L. J. Palkovacs E. P. Post D. M. Therkildsen N. O. Flecker A. S. (2022). The evolutionary consequences of dams and other barriers for riverine fishes. BioScience 72, 431–448. 10.1093/biosci/biac004

[B90] Zeng J. Jiao C. Zhao D. Xu H. Huang R. Cao X.. (2019). Patterns and assembly processes of planktonic and sedimentary bacterial community differ along a trophic gradient in freshwater lakes. Ecol. Indicators. 106:105491. 10.1016/j.ecolind.2019.105491

[B91] Zhang B. Zhang J. Liu Y. Shi P. Wei G. (2018). Co-occurrence patterns of soybean rhizosphere microbiome at a continental scale. Soil Biol. Biochem. 118, 178–186. 10.1016/j.soilbio.2017.12.011

[B92] Zhang J. Yang Y. Zhao L. Li Y. Xie S. Liu Y.. (2015). Distribution of sediment bacterial and archaeal communities in plateau freshwater lakes. Appl. Microbiol. Biotechnol. 99, 3291–3302. 10.1007/s00253-014-6262-x 25432677

[B93] Zhang X. J. Chen C. Ding J. Q. Hou A. Li Y. Niu Z. B.. (2010). The 2007 water crisis in Wuxi, China: analysis of the origin. J. Hazard. Mater. 182, 130–135. 10.1016/j.jhazmat.2010.06.006 20591562

[B94] Zhou H. Gao Y. Jia X. Wang M. Ding J. Cheng L.. (2020). Network analysis reveals the strengthening of microbial interaction in biological soil crust development in the Mu Us Sandy Land, northwestern China. Soil Biol. Biochem. 144:107782. 10.1016/j.soilbio.2020.107782

[B95] Zhou J. Ning D. (2017). Stochastic community assembly: does it matter in microbial ecology? Microbiol. Mol. Biol. Rev. 81, e00002–e00017. 10.1128/MMBR.00002-17 29021219 PMC5706748

[B96] Zuo J. Xiao P. Heino J. Tan F. Soininen J. Chen H.. (2024). Eutrophication increases the similarity of cyanobacterial community features in lakes and reservoirs. Water Res. 250:120977. 10.1016/j.watres.2023.120977 38128306

