# Supplementary material for: Sediment bacterial biogeography across reservoirs in the Hanjiang river basin, southern China: the predominant influence of eutrophication-induced carbon enrichment
Source: Front Microbiol. 2025 Mar 28;16:1554914. doi: 10.3389/fmicb.2025.1554914 (PMC11991844; doi:10.3389/fmicb.2025.1554914)
Supplement: Supplementary file 1 [file Table_1.docx]

**Table S1** Spearman's rho rank correlations among pairwise environmental factors. TC, sediment total carbon; SWC, sediment water content; TS, sediment total sulfur; TH, sediment total hydrogen; Ca^2+^, sediment calcium; Fe^3+^, sediment iron; Cu^2+^, sediment copper; Mn^2+^, sediment manganese; TP, sediment total phosphorus; TN, sediment total nitrogen; TSI, water trophic state index; Chla, water chlorophyll *a*.

|  | TC | TH | SWC | Mg^2+^ | Ca^2+^ | Fe^3+^ | Cu^2+^ | TS | Mn^2+^ | TP | TN | TSI | Chla |
| --- | --- | --- | --- | --- | --- | --- | --- | --- | --- | --- | --- | --- | --- |
| TC |  |  |  |  |  |  |  |  |  |  |  |  |  |
| TH | 0.544*** |  |  |  |  |  |  |  |  |  |  |  |  |
| SWC | 0.83*** | 0.499*** |  |  |  |  |  |  |  |  |  |  |  |
| Mg^2+^ | -0.077 | -0.235 | 0.133 |  |  |  |  |  |  |  |  |  |  |
| Ca^2+^ | -0.184 | -0.272* | -0.087 | 0.749*** |  |  |  |  |  |  |  |  |  |
| Fe^3+^ | 0.027 | -0.163 | 0.198 | 0.785*** | 0.625*** |  |  |  |  |  |  |  |  |
| Cu^2+^ | 0.1 | 0.152 | 0.216 | 0.264* | 0.192 | 0.219 |  |  |  |  |  |  |  |
| TS | 0.464*** | 0.427*** | 0.469*** | 0.273* | 0.237 | 0.346** | 0.275* |  |  |  |  |  |  |
| Mn^2+^ | -0.09 | -0.005 | -0.014 | 0.431*** | 0.356** | 0.21 | 0.558*** | 0.197*** |  |  |  |  |  |
| TP | 0.681*** | 0.389** | 0.774*** | 0.153 | -0.029 | 0.275* | 0.306* | 0.495** | 0.113 |  |  |  |  |
| TN | 0.489 | 0.242 | 0.444*** | 0.135 | 0.046 | 0.27 | 0.139 | 0.378 | 0.068 | 0.601*** |  |  |  |
| TSI | 0.405*** | 0.209 | 0.341** | -0.127 | 0.076 | 0.138 | 0.147 | 0.301* | 0.05 | 0.514*** | 0.301* |  |  |
| Chla | 0.426*** | 0.128 | 0.512*** | 0.089 | 0.118 | 0.394** | 0.133 | 0.347** | -0.028 | 0.532*** | 0.379** | 0.737*** |  |

*: *p* < 0.05; **: *p* < 0.01.
