# Supplementary material for: Sediment bacterial biogeography across reservoirs in the Hanjiang river basin, southern China: the predominant influence of eutrophication-induced carbon enrichment
Source: Front Microbiol. 2025 Mar 28;16:1554914. doi: 10.3389/fmicb.2025.1554914 (PMC11991844; doi:10.3389/fmicb.2025.1554914)
Supplement: Supplementary file 2 [file Table_2.docx]

**Table S2** The α-diversity of sediment bacterial community in reservoirs of the Hanjiang River Basin, southern China. The locations and the full names of the studied reservoirs are given below.

| Index | Longitude | Latitude | Richness | Shannon | Simpson | Pielou | Chao1 | ACE | Coverage |
| --- | --- | --- | --- | --- | --- | --- | --- | --- | --- |
| Changtan reservoir (CT) | 116.115° | 24.726° | 7757 | 11.182 | 0.998 | 0.865 | 10108 | 10289 | 0.954 |
| Duobao reservoir (DB) | 116.363° | 24.857° | 7456 | 10.995 | 0.998 | 0.855 | 9735 | 9933 | 0.957 |
| Dongfanghong reservoir (DFH) | 115.695° | 23.893° | 6606 | 10.539 | 0.996 | 0.831 | 8848 | 9093 | 0.953 |
| Fenghuang reservoir (FH) | 116.663° | 23.901° | 7550 | 11.106 | 0.998 | 0.862 | 9765 | 9930 | 0.957 |
| Fushi reservoir (FS) | 115.803° | 24.445° | 7689 | 11.188 | 0.998 | 0.867 | 9990 | 10185 | 0.953 |
| Fengxi reservoir (FX) | 116.768° | 23.815° | 6486 | 11.051 | 0.999 | 0.873 | 8487 | 8752 | 0.950 |
| Gangshan reservoir (GS) | 116.738° | 23.660° | 6597 | 10.651 | 0.996 | 0.839 | 8684 | 8823 | 0.956 |
| Guitian reservoir (GT) | 115.702° | 24.259° | 6959 | 11.104 | 0.999 | 0.870 | 9294 | 9510 | 0.947 |
| Heshui reservoir (HS) | 115.838° | 23.822° | 7082 | 11.065 | 0.998 | 0.865 | 9289 | 9541 | 0.953 |
| Heshanyan reservoir (HSY) | 115.748° | 24.256° | 5330 | 10.082 | 0.994 | 0.815 | 7281 | 7477 | 0.953 |
| Huangtian reservoir (HT) | 115.881° | 24.706° | 7155 | 10.980 | 0.997 | 0.858 | 9246 | 9454 | 0.956 |
| Huangzhuping reservoir (HZP) | 116.239° | 24.729° | 6859 | 10.896 | 0.998 | 0.855 | 8917 | 9049 | 0.958 |
| Mianhuatan reservoir (MHT) | 116.527° | 24.415° | 7067 | 10.916 | 0.998 | 0.854 | 9305 | 9530 | 0.953 |
| Meixi reservoir (MX) | 115.937° | 24.377° | 7024 | 10.995 | 0.998 | 0.861 | 9211 | 9367 | 0.957 |
| Pengxi reservoir (PX) | 116.693° | 23.954° | 7823 | 11.367 | 0.999 | 0.879 | 10110 | 10261 | 0.957 |
| Qingliangshan reservoir (QLS) | 116.234° | 24.204° | 6157 | 10.696 | 0.998 | 0.850 | 8059 | 8230 | 0.963 |
| Qingxi reservoir (QX) | 116.571° | 24.675° | 6253 | 10.557 | 0.997 | 0.837 | 7958 | 8043 | 0.969 |
| Shibi reservoir (SB) | 115.787° | 24.187° | 5816 | 10.503 | 0.997 | 0.840 | 7690 | 7826 | 0.960 |
| Wengong reservoir (WG) | 115.802° | 24.557° | 6445 | 10.854 | 0.998 | 0.858 | 8463 | 8546 | 0.957 |
| Yanqian reservoir (YQ) | 115.901° | 23.895° | 5633 | 10.306 | 0.994 | 0.827 | 7122 | 7199 | 0.970 |
| Yitang reservoir (YT) | 115.614° | 23.946° | 5487 | 10.290 | 0.997 | 0.829 | 7100 | 7172 | 0.970 |
