# Supplementary material for: Sediment bacterial biogeography across reservoirs in the Hanjiang river basin, southern China: the predominant influence of eutrophication-induced carbon enrichment
Source: Front Microbiol. 2025 Mar 28;16:1554914. doi: 10.3389/fmicb.2025.1554914 (PMC11991844; doi:10.3389/fmicb.2025.1554914)
Supplement: Supplementary file 3 [file Image_1.pdf]

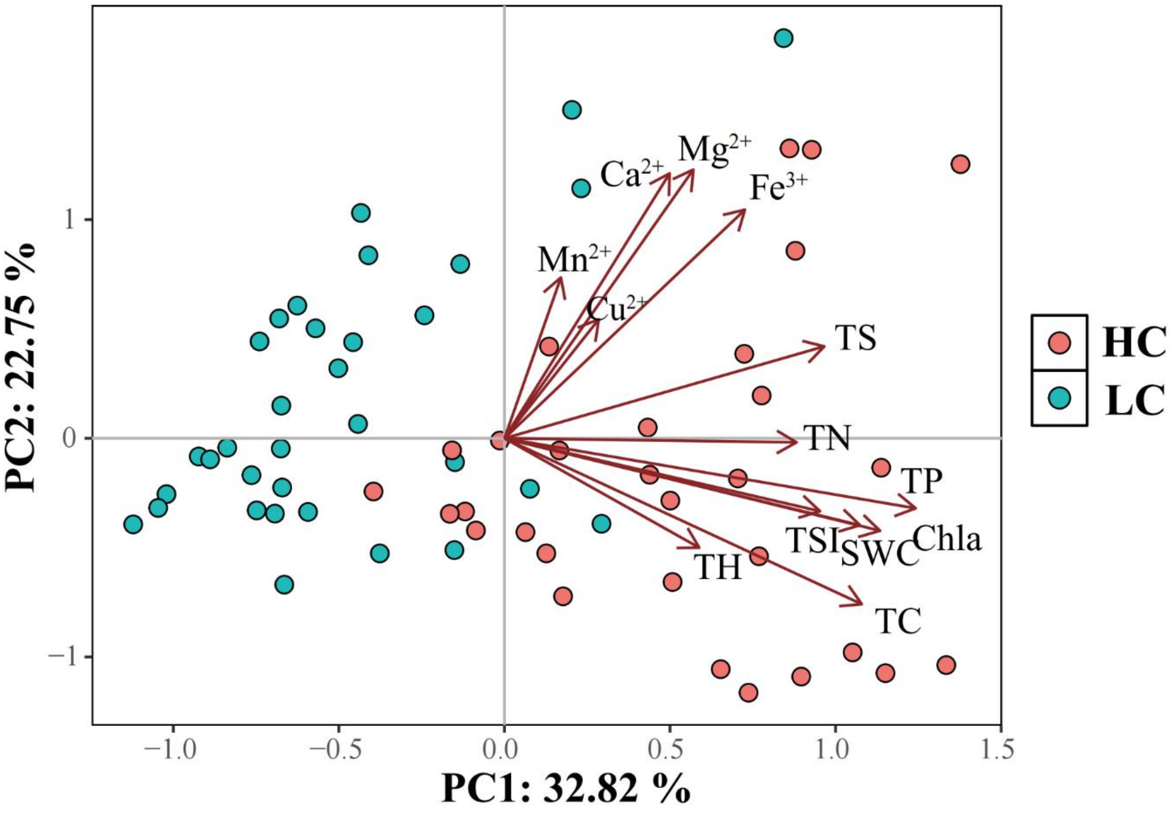

FIGURE S1 Principal component analysis (PCA) depicting the relationships among all of the investigated environmental variables. TC, sediment total carbon; SWC, sediment water content; TS, sediment total sulfur; TH, sediment total hydrogen; Ca^{2+}, sediment calcium; Fe^{3+}, sediment iron; Cu^{2+}, sediment copper; Mn^{2+}, sediment manganese; TP, sediment total phosphorus; TN, sediment total nitrogen; TSI, water trophic state index; Chla, water chlorophyll *a*. HC: high-carbon reservoirs (TC content ≥ 13.2 $g \cdot kg^{-1}$), LC: low-carbon reservoirs (TC content < 13.2 $g \cdot kg^{-1}$).
