# Supplementary material for: Sediment bacterial biogeography across reservoirs in the Hanjiang river basin, southern China: the predominant influence of eutrophication-induced carbon enrichment
Source: Front Microbiol. 2025 Mar 28;16:1554914. doi: 10.3389/fmicb.2025.1554914 (PMC11991844; doi:10.3389/fmicb.2025.1554914)
Supplement: Supplementary file 4 [file Image_2.pdf]

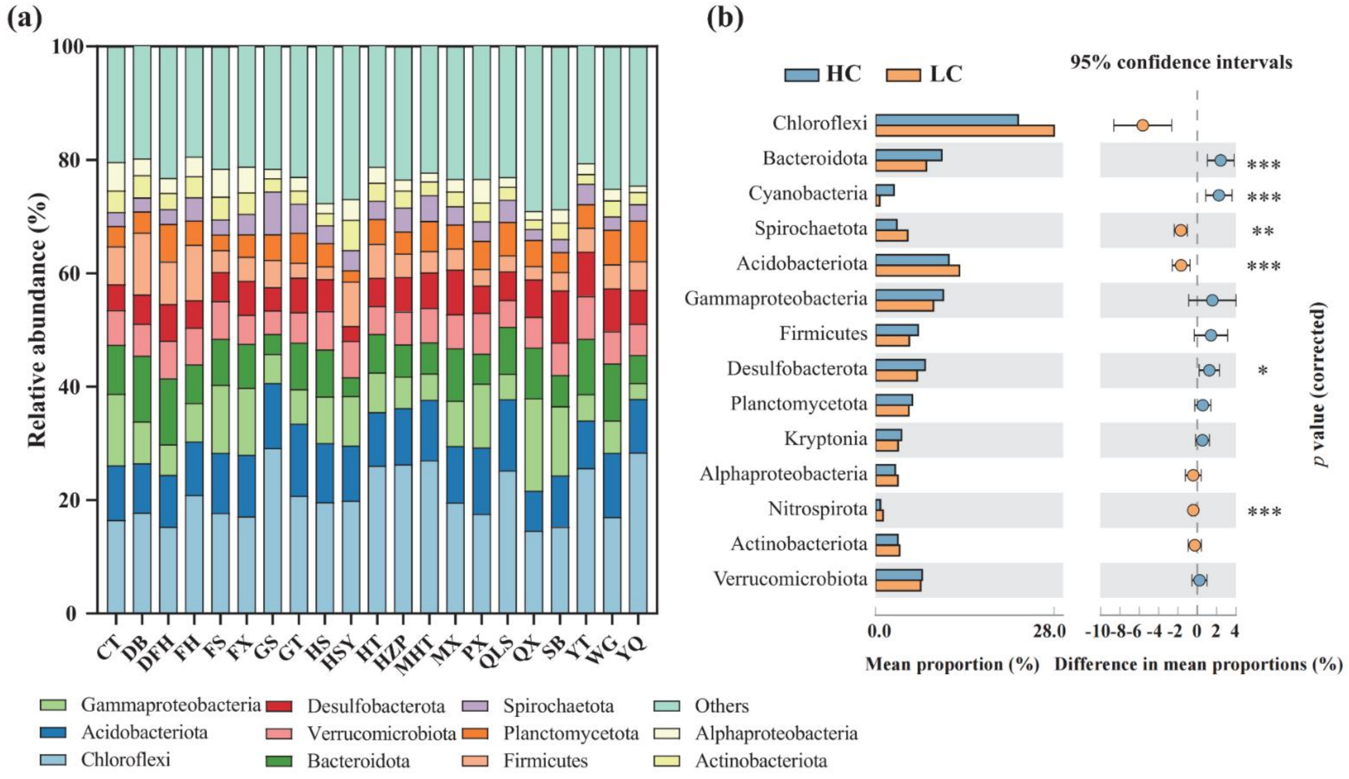

FIGURE S2. The compositions of sediment bacterial communities along a total carbon gradient (a) and STAMP analysis of significant differences in bacterial community compositions between high-carbon and low-carbon groups (b). HC: high-carbon reservoirs (TC content ≥ 13.2 $g \cdot kg^{-1}$), LC: low-carbon reservoirs (TC content < 13.2 $g \cdot kg^{-1}$).
