# Supplementary material for: Sediment bacterial biogeography across reservoirs in the Hanjiang river basin, southern China: the predominant influence of eutrophication-induced carbon enrichment
Source: Front Microbiol. 2025 Mar 28;16:1554914. doi: 10.3389/fmicb.2025.1554914 (PMC11991844; doi:10.3389/fmicb.2025.1554914)
Supplement: Supplementary file 5 [file Image_3.pdf]

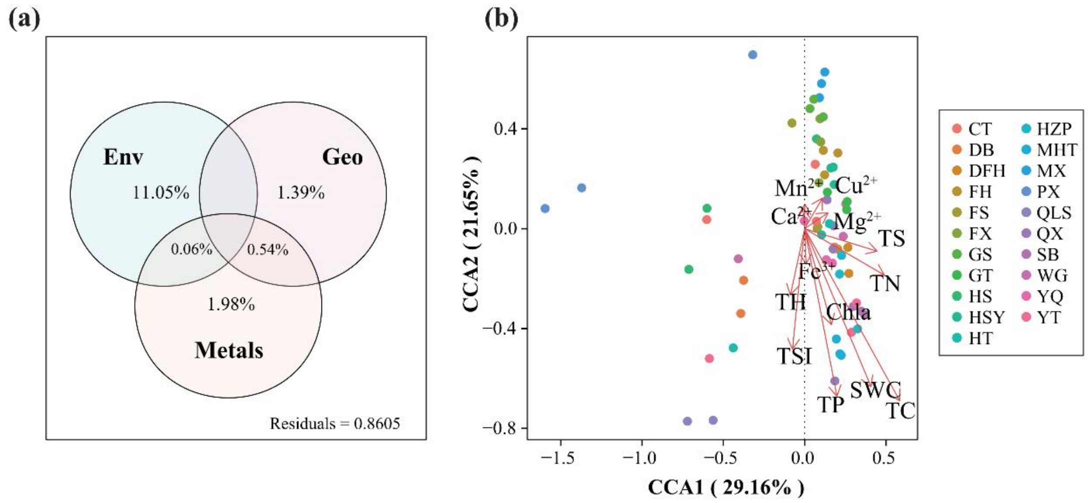

FIGURE S3 (a) The relationships between sediment bacteria community compositions and the best subset of sediment environmental factors (env: TC, TP, TN and SWC), metal ions (Metals: Ca^{2+}, Mg^{2+}, Fe^{3+}, Cu^{2+} and Mn^{2+}), and geographical parameters in variance partitioning analysis (VPA); (b) Canonical correspondence analysis (CCA) relating bacterial community compositions with sediment environmental variables. Points in the graph represent the sampling sites. The length and angle of red arrows represent the impact of environmental factors on bacterial community structure. TC, sediment total carbon; SWC, sediment water content; TS, sediment total sulfur; TH, sediment total hydrogen; Ca^{2+}, sediment calcium; Fe^{3+}, sediment iron; Cu^{2+}, sediment copper; Mn^{2+}, sediment manganese; TP, sediment total phosphorus; TN, sediment total nitrogen; TSI, water trophic state index; Chla, water chlorophyll *a*.
