# Supplementary material for: Sediment bacterial biogeography across reservoirs in the Hanjiang river basin, southern China: the predominant influence of eutrophication-induced carbon enrichment
Source: Front Microbiol. 2025 Mar 28;16:1554914. doi: 10.3389/fmicb.2025.1554914 (PMC11991844; doi:10.3389/fmicb.2025.1554914)
Supplement: Supplementary file 6 [file Image_4.pdf]

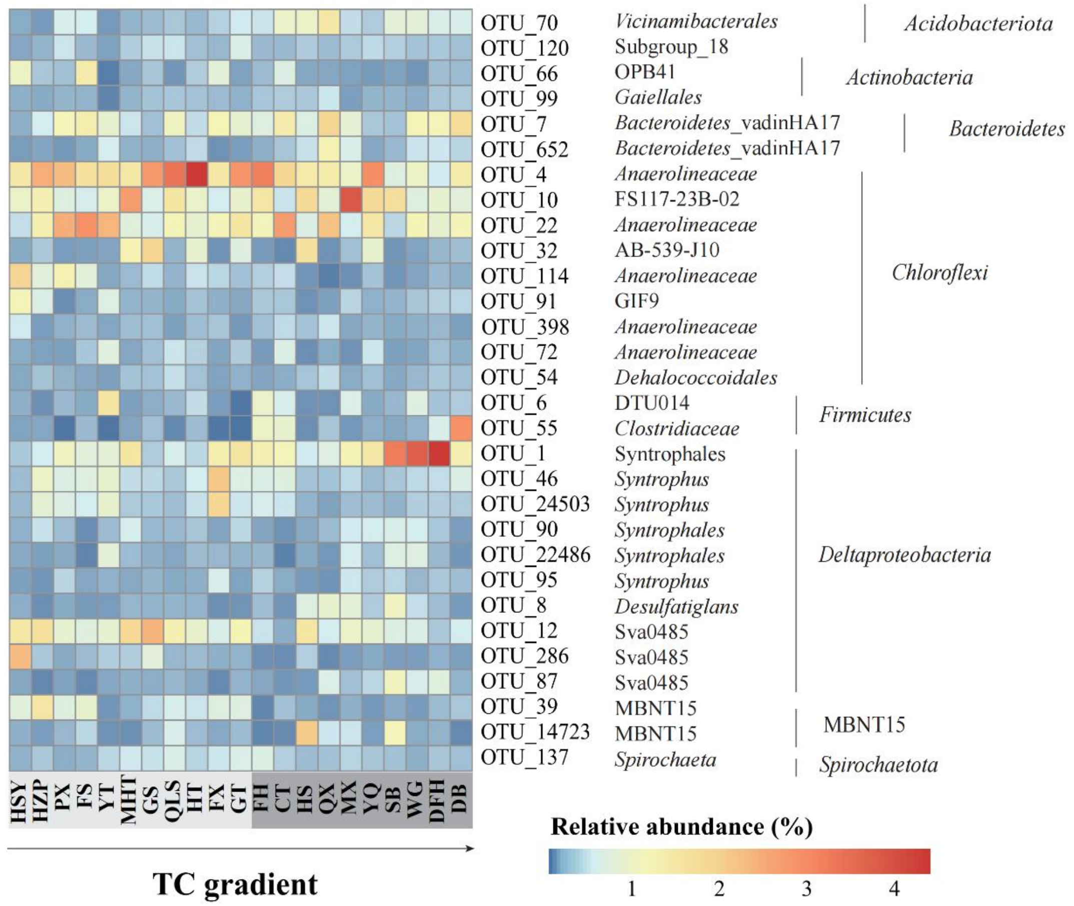

FIGURE S4 Heat map describing the relative abundance of the top 30 OTU bacterial lineages or clades of sediment bacterial communities (Y axis) across the total carbon gradient (X axis). High heterogeneity in community compositions was also evident at the lineage and clade levels along the total carbon gradient.
